# A quantitative brain map of experimental cerebral malaria pathology

**DOI:** 10.1371/journal.ppat.1006267

**Published:** 2017-03-08

**Authors:** Patrick Strangward, Michael J. Haley, Tovah N. Shaw, Jean-Marc Schwartz, Rachel Greig, Aleksandr Mironov, J. Brian de Souza, Sheena M. Cruickshank, Alister G. Craig, Danny A. Milner, Stuart M. Allan, Kevin N. Couper

**Affiliations:** 1 Faculty of Biology, Medicine and Health, University of Manchester, Manchester, United Kingdom; 2 Immunology Unit, Department of Infectious and Tropical Diseases, London School of Hygiene and Tropical Medicine, London, United Kingdom; 3 Department of Molecular and Biochemical Parasitology, Liverpool School of Tropical Medicine, Liverpool, United Kingdom; 4 Department of Pathology, The Brigham & Women’s Hospital, Boston, Massachusetts, United States of America; Queensland Institute of Medical Research, AUSTRALIA

## Abstract

The murine model of experimental cerebral malaria (ECM) has been utilised extensively in recent years to study the pathogenesis of human cerebral malaria (HCM). However, it has been proposed that the aetiologies of ECM and HCM are distinct, and, consequently, no useful mechanistic insights into the pathogenesis of HCM can be obtained from studying the ECM model. Therefore, in order to determine the similarities and differences in the pathology of ECM and HCM, we have performed the first spatial and quantitative histopathological assessment of the ECM syndrome. We demonstrate that the accumulation of parasitised red blood cells (pRBCs) in brain capillaries is a specific feature of ECM that is not observed during mild murine malaria infections. Critically, we show that individual pRBCs appear to occlude murine brain capillaries during ECM. As pRBC-mediated congestion of brain microvessels is a hallmark of HCM, this suggests that the impact of parasite accumulation on cerebral blood flow may ultimately be similar in mice and humans during ECM and HCM, respectively. Additionally, we demonstrate that cerebrovascular CD8^+^ T-cells appear to co-localise with accumulated pRBCs, an event that corresponds with development of widespread vascular leakage. As in HCM, we show that vascular leakage is not dependent on extensive vascular destruction. Instead, we show that vascular leakage is associated with alterations in transcellular and paracellular transport mechanisms. Finally, as in HCM, we observed axonal injury and demyelination in ECM adjacent to diverse vasculopathies. Collectively, our data therefore shows that, despite very different presentation, and apparently distinct mechanisms, of parasite accumulation, there appear to be a number of comparable features of cerebral pathology in mice and in humans during ECM and HCM, respectively. Thus, when used appropriately, the ECM model may be useful for studying specific pathological features of HCM.

## Author summary

Cerebral malaria (HCM) is the most severe complication of malaria infection. Despite this, we have an incomplete understanding of the cause (pathogenesis) of the syndrome. To improve our understanding of HCM pathogenesis, animal models of the syndrome have been developed. The most commonly used model is the murine experimental cerebral malaria (ECM) model. However, to date, there has not been a detailed investigation of the pathology of ECM using the same methodological approaches (histopathology) employed in the study of HCM. Thus, it has been unclear whether ECM is a valid model for HCM. In this histopathological study, we show that, as in HCM, cerebrovascular parasite accumulation is an important feature of ECM. However, unlike HCM, we did not observe large numbers of parasitised red blood cells (pRBCs) attached to the walls of cerebral blood vessels during ECM; instead individual pRBCs were trapped in narrow murine brain capillaries. Nevertheless, despite this, we showed that cerebrovascular parasites were still associated with disturbed blood flow, vascular leakage and impaired neuronal function in ECM, in a similar fashion to that reported in HCM. Therefore, our results define the specific aspects of HCM pathology that can potentially be studied within the ECM model.

## Introduction

Cerebral Malaria (CM), one of the most severe complications of *Plasmodium falciparum* (*Pf*) infection, is defined clinically by an unrousable coma in the presence of *Pf* parasitemia, with no other known cause of neuropathology [1]. Although the syndrome only occurs in 1% of *Pf* infections, it has a high fatality rate (15–20% of cases), with death typically occurring despite administration of established anti-malarial drug regimens [1, 2]. Moreover, whilst CM induced-encephalopathy has historically been considered acute and reversible, recent follow-up studies in individuals post-CM have determined that a significant percentage (10–26%) exhibit long-term neurological sequelae [3]. Individuals with limited prior exposure to parasite are disproportionally susceptible to the syndrome [4]; as a result, the majority of fatal CM cases consist of young children in endemic regions of Africa [5]. Indeed, with an estimated 2–3 million cases of the syndrome annually, CM-associated mortality and neuro-disability imposes a substantial social and economic burden on this region [6, 7]. Consequently, there remains an urgent need to understand the pathogenesis of CM, to facilitate the development of more efficacious anti-malarial drugs and/or adjunct therapies for the condition.

Neuropathological studies from fatal CM cases have detailed dense sequestration of parasitised erythrocytes (pRBCs) within the cerebral micro-vasculature as a canonical feature of the syndrome [8–11]. Indeed, pRBC sequestration in cerebral capillaries and venules is quantitatively greater in HCM patients, than in individuals who succumb to non-cerebral malarial complications [9, 10]. It is believed that pRBC congestion of vessels may impair tissue perfusion by perturbing cerebral flow, and/or lead to local immune-mediated injury via secondary host response(s) to parasite products [11, 12]. However, accumulating evidence indicates that CM is a relatively complex neuropathology, with pRBC sequestration typically occurring concomitant with significant intravascular accumulation of mononuclear cells, intracerebral haemorrhage, enhanced blood-brain barrier (BBB) permeability and oedema [5]. Moreover, pathology is not restricted to the cerebral vasculature during CM, and axonal injury and demyelination have also been documented [5, 13]. Nevertheless, despite our knowledge of the pathology of fatal CM, restricted access to post-mortem samples for histopathological study has prevented correlation of pathological features with onset of clinical symptoms. Thus, the importance and/or relative contributions of the above observed pathological events to pathogenesis of CM remains incompletely understood.

Inaccessibility of the human brain pre-mortem has led to the development and study of the experimental mouse model of cerebral malaria (ECM) [14–20]. Susceptible mice infected with *Plasmodium berghei* (*Pb*) ANKA present with similar graded and sequential signs of disease as humans affected with CM (HCM); including ataxia, paralysis, coma and, if untreated, death [21]. Furthermore, mice treated with anti-malarial drugs at the point of neurological dysfunction demonstrate comparable levels of mortality and long-term cognitive dysfunction [22, 23]. Such similarities in clinical presentation and long-term consequence between ECM & HCM, suggest the pathophysiological processes underlying the two conditions may be comparable. Indeed, parasite accumulation has been observed in the brains of mice that developed fatal malaria-induced cerebral pathology compared with those that developed asymptomatic infections [24]. Concurrent to parasite accumulation, haemorrhage and BBB disruption have also been observed in the brains of mice experiencing ECM; with the latter perceived as a key feature of the syndrome [25, 26]. Additionally, brain-accumulating CD8^+^ T-Cells have been shown to play a critical role in ECM pathogenesis by promoting BBB disruption via perforin and Granzyme B dependent mechanisms [27–29], potentially following interaction with brain endothelial cells cross-presenting parasite antigen [30, 31].

Despite the extensive use of the ECM model, there remains significant debate regarding its validity to study HCM [21, 32–35]. In particular, the importance of pRBC sequestration within the brain for the development of ECM has been questioned [36]. Indeed, it is currently unknown whether true pRBC sequestration occurs during ECM, or if pRBCs simply accumulate within intracerebral haemorrhages and/or leukocyte occluded brain vessels [37]. This lack of understanding is because intracerebral parasite accumulation during ECM has previously been studied using spatially insensitive techniques such as RT-PCR or whole body luminescent imaging, rather than through detailed histopathological assessment, such as performed during HCM [24, 36–39]. Importantly, the lack of detailed histopathological knowledge of the ECM syndrome means we also currently do not know the spatial relationship between pRBCs and other pathological parameters involved in ECM development, such as CD8^+^ T-cells, haemorrhage and oedema, or how these pathological events affect different brain regions. Consequently, at present the pathology of ECM has been incompletely characterised and, as such, it is not possible to definitively conclude whether it is a valid model to study all, some, or none of the pathological features of HCM.

In this study, to improve our knowledge of the pathology of ECM, we have performed a systematic and quantitative histopathological investigation of ECM using comparable methodologies as utilised in the study of HCM. Crucially, we show that intracapillary parasite accumulation throughout the brain is a canonical feature of ECM, and that a single mature, pRBC seems sufficient to occlude narrow murine capillaries, and thus cause localised haemostasis. Parasite accumulation also appears to mediate the subsequent local intravascular recruitment of low numbers of CD8^+^ T-cells that, together with parasite, is associated with widespread BBB disruption. Strikingly, BBB disruption appears to occur due to vascular junction remodelling and increased levels of caveolae, rather than through extensive endothelial cell apoptosis. Finally, we detected axonal and myelin injury adjacent to multiple neurovascular pathogenic parameters associated with ECM, indicating two potential common pathways for neurological impairment to occur during malaria-induced cerebral pathology. Collectively, our data indicates that the mechanisms underlying the response of the brain to local parasite accumulation are conserved between humans and mice, and, therefore, supports the use of the ECM model to understand the pathogenesis of HCM.

## Results

### The course of blood stage *Pb* ANKA (ECM-inducing) and *Pb* NK65 (uncomplicated) infections

**Fig 1:**
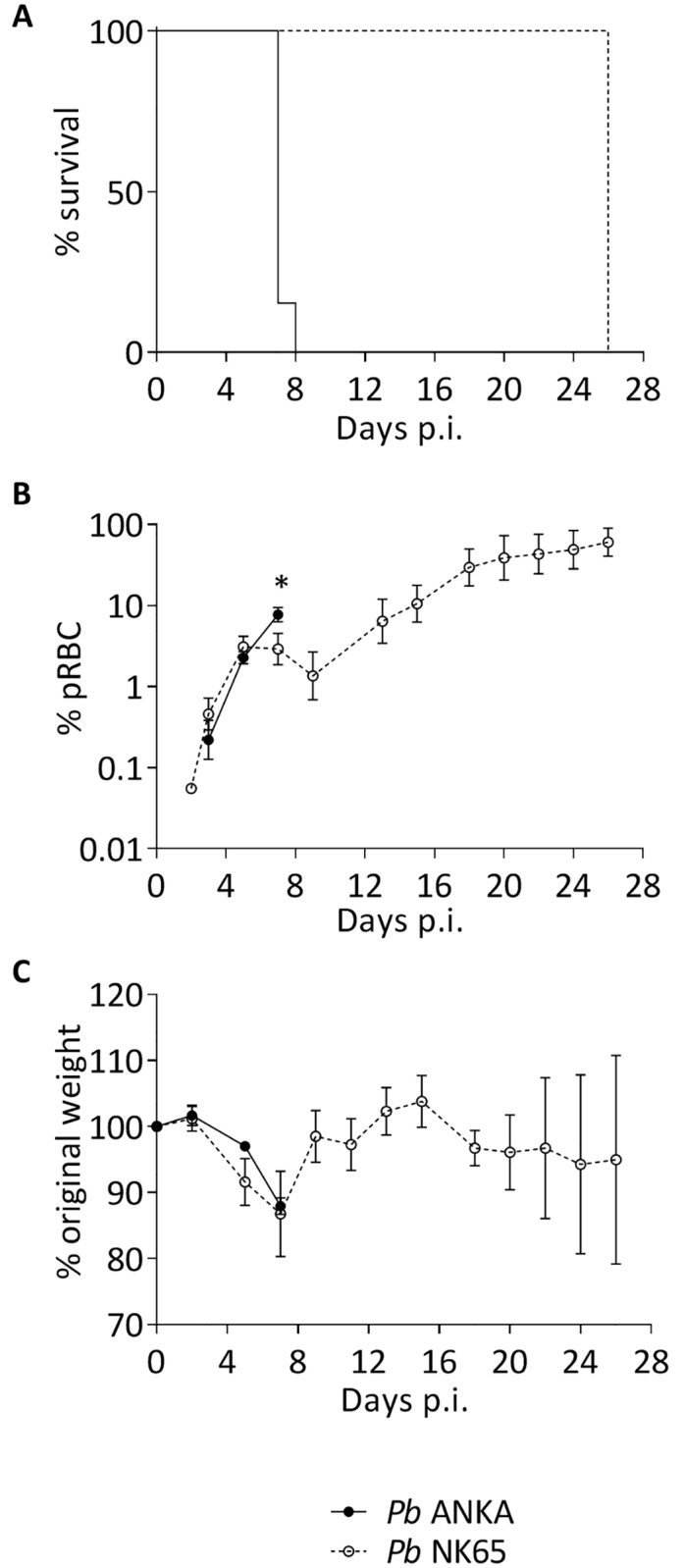
Survival, parasitemia and weight loss during the course of blood stage *Pb* ANKA and *Pb* NK65 infection. C57BL/6 mice were infected with 1x10^4^ *Pb* ANKA GFP (n = 13) or 1x10^4^ *Pb* NK65 GFP (n = 12) pRBCs. **(A)** Survival was monitored daily during the window period (days 6–12 p.i.) for ECM, and **(B)** peripheral parasitemia ±SEM and **(C)** weight loss ±SD were monitored every other day during the course of infection.

We sought to characterise the pathological features specifically associated with ECM, compared with those that simply occur during uncomplicated malaria infection. Therefore, we utilised two closely related murine plasmodium strains with contrasting infection outcomes. Consistent with our previous studies [26, 40, 41], C57BL/6 mice infected with *Pb* ANKA typically developed signs of late stage ECM; including ataxia, convulsions, paralysis and/or coma, on day 7 (p.i.) (Fig 1A). In contrast, mice infected with *Pb* NK65, despite exhibiting comparable parasitemia (with the notable exception of day 7 p.i.) (Fig 1B) and weight loss (Fig 1C) as mice infected with *Pb* ANKA, survived the critical window for developing ECM (days 6–12 p.i.) without exhibiting neurological symptoms. *Pb* NK65 infected mice instead developed hyperparasitemia and succumbed to infection on day 25 (Fig 1A). Thus, this comparative model provides a tractable way to identify host and parasitological events that specifically contribute to the development and progression of malaria-induced cerebral pathology.

### Intracerebral pRBC sequestration during *Pb* infections

**Fig 2:**
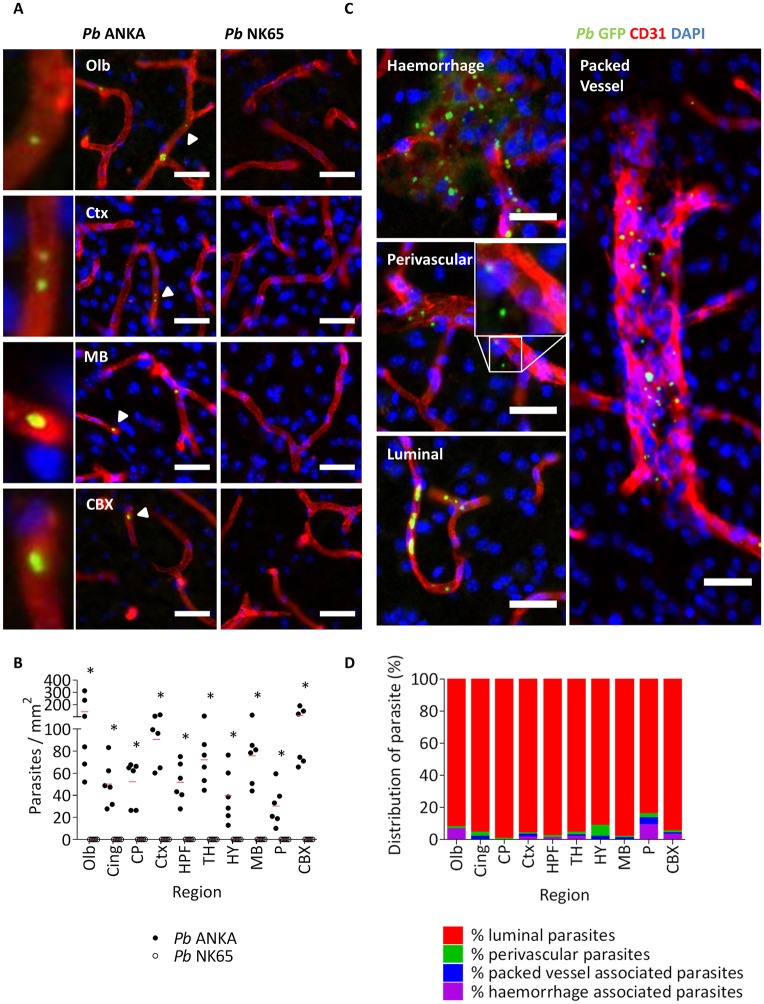
Intracerebral parasite accumulation occurs exclusively during ECM infection. C57BL/6 mice were infected with 1x10^4^ *Pb* ANKA GFP (n = 6) or 1x10^4^ *Pb* NK65 GFP (n = 6) pRBCs. Mice were culled on d7 p.i. when *Pb* ANKA infected mice exhibited signs of late-stage ECM Brains were removed from transacardially perfused mice and examined via immunofluorescence for the presence of GFP+ pRBCs (green) in relation to CD31+ vasculature (red), with nuclei counterstained blue. **(A)** Representative images demonstrate the presence of GFP+ parasites (Δ) in the olfactory bulbs, cortex, midbrain and cerebellar cortex of *Pb* ANKA infected mice, and their respective absence in *Pb* NK65 infected mice. Identified parasite is highlighted in digitally magnified panel adjacent (left). **(B)** Quantitation of GFP+ parasites within the different brain regions of *Pb* ANKA and *Pb* NK65 infected mice. **(C)** Representative images of the different types of cerebral parasite accumulation that occur during ECM. **(D)** The frequency (%) of GFP+ parasite observed to be: luminal; perivascular; haemorrhage-associated; or packed-vessel associated. Dots represent mean of individual brains, with red lines mean of total brains. Bars represent mean frequency for all brains. Scale bar: 25μm. *p ≤ 0.05 (unpaired t-test, no assumption made for consistent SD).

Although analyses using RT-PCR and luciferase-expressing parasites have shown that ECM is associated with accumulation of parasites in the brain [24, 37–39], the compartmentalisation of the pRBCs in the brain during ECM is not known. Consequently, whether parasite sequestration, and subsequent microvascular obstruction, occurs within the brain during ECM development is, at present, unclear. Therefore, we performed a detailed analysis of parasite accumulation in the well-perfused brains of mice infected with *Pb* ANKA and *Pb* NK65. Utilising GFP-tagged parasites, which enabled us to perform high resolution histopathological analyses of pRBC location within the intact brain architecture, we observed significantly higher accumulation of pRBCs in all assessed brain regions (S1 Fig) during *Pb* ANKA infection compared with *Pb* NK65 infection (we did not detect any innate signal through the GFP channel in the brains of uninfected mice) (Fig 2A and 2B & S2 Fig). We noted differences in the size of parasite GFP signal, and confirmed these differences in expression related to parasite maturity using whole brain homogenate (S3 Fig). To exclude the possibility that differences in GFP expression by Pb ANKA and Pb NK65 parasites were responsible for the observed differences in parasite accumulation, we additionally confirmed that significantly greater intracerebral parasite accumulation occurs during *Pb* ANKA infection compared to *Pb* NK65 infection by utilising *Pb* anti-sera (S4 Fig). Whilst GFP expression is constrained to live parasite, *Pb* anti-sera visualised all parasite material, and thus the degree of *Pb* anti-sera immunoreactivity was much higher relative to anti-GFP staining from the same samples (S4 Fig). Importantly, we noted that the >90% of intracerebral pRBCs during ECM were intracapillary, rather than associated with intravascular accumulations of leukocytes and/or haemorrhage (Fig 2C and 2D). Combined, these results demonstrate that global parasite accumulation within cerebral capillaries is a specific event associated with ECM.

**Fig 3:**
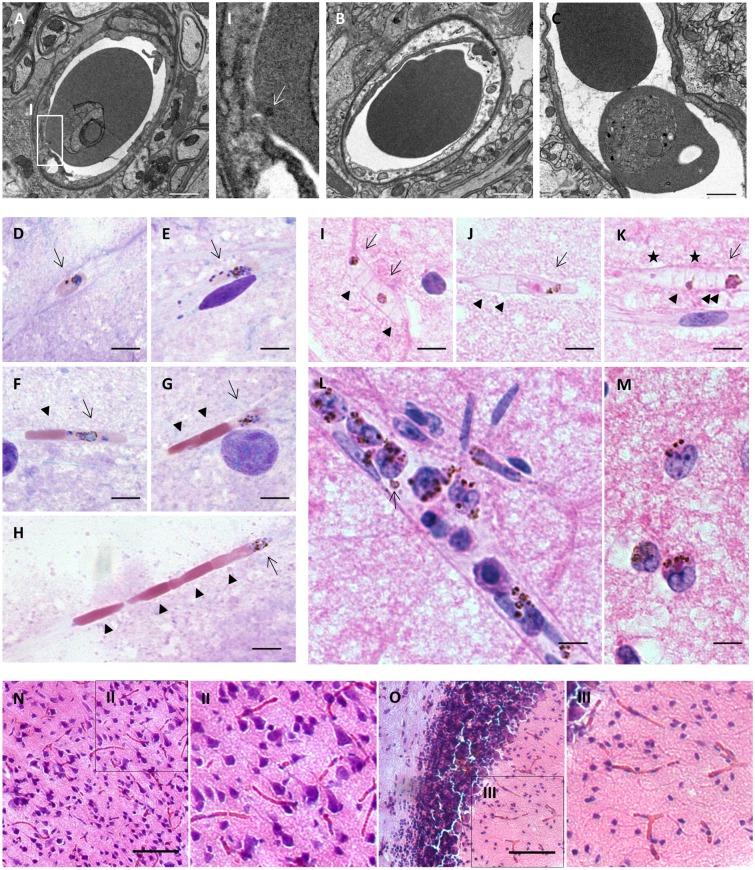
A single, pRBC is sufficient to occlude a capillary in the brains of mice infected with *Pb* ANKA. C57BL/6 mice were infected with 1x10^4^ Pb ANKA GFP pRBCs and euthanised upon developing ECM (d7 p.i.) Transcardially perfused brains were dissected out and processed for TEM **(Figs A-C)** (n = 3), smeared for cytological examination **(Figs D-M)** (n = 5) or processed for histological investigation **(Figs N-O)** (n = 8). Electron micrographs of: **(A)** a sequestered pRBC in the cross-section of a capillary, **(i)** with the adjacent digitally magnified panel highlighting an electron-dense spot (↑) on the infected erythrocyte surface, proximal to the endothelium. **(B)** An uninfected, highly deformed RBC squeezing through a capillary. **(C)** An uninfected RBC trapped behind a sequestered pRBC in a capillary longitudinal-section. Brain smears stained via Quik-Diff depicting: a **(D)** mid to late-stage trophozoite (↑) and a **(E)** late-stage schizont (↑) sequestered within capillaries. Capillaries with **(F)** 1, **(G)** 2 and **(H)** 4 uninfected RBCs (▲) trapped behind a single, mature pRBC (↑). H&E stained brain smears demonstrating: **(I)** uninfected erythrocytes (▲), trapped behind cytoadherent pigmented pRBCs (↑), deformed by the narrow capillary lumen. **(J)** A pigmented, mid-stage trophozoite (↑) sequestered within a capillary entraps two uninfected RBCs (▲). **(K)** A sequestered schizont (↑) occludes a capillary, with a “tail” of uninfected RBCs (▲) and immature pRBCs (★) behind it. **(L)** A larger calibre vessel, congested primarily by macrophages dense with parasite material. Extra-erythrocytic parasite (↑) is associated with macrophage, rather than endothelium. **(M)** Extravascular macrophage enriched with parasite material. 30μm thick, conventional histological sections stained for H&E demonstrate extensive erythrocytic accumulation (in which pRBCs are a minority) in both **(N)** cortical and **(O)** cerebellar grey matter, also shown **(ii, iii)** at higher magnification in adjacent panels.. Scale bars: A-C 1μm; D-M 5μm; N and O 50μm.

As pRBC accumulation is principally compartmentalised within the microvasculature during ECM, we theorised that this phenomenon is likely dependent on a form of sequestration. Accordingly, we utilised transmission electron microscopy (TEM) to characterise the precise nature of the interaction between pRBCs and cerebral endothelial cells (ECs) in the brains of mice infected with *Pb* ANKA. Consistent with our immunofluorescence staining, we observed pRBCs within the capillaries of perfused brains from *Pb* ANKA mice with late-stage ECM (Fig 3A). Moreover, we detected the occasional electron-dense spot on the surface of some pRBCs adjacent to the EC membrane (Fig 3AI); such events were not evident on the surface of uninfected erythrocytes (Fig 3B). Whilst we noted greater numbers of luminal RBCs compared to luminal pRBCs, importantly, longitudinal sections of capillaries invariably demonstrated that such RBCs accumulated specifically behind vessel-spanning pRBCs (Fig 3C). We further assessed the level and presentation of intravascular pRBC accumulation during ECM by smearing the well-perfused brain tissue of mice infected with *Pb* ANKA. Brain smears preserve lengthy microvessels, and thus have historically been used in HCM for assessing sequestration [42, 43]. We observed several intracapillary trophozoites and schizonts in Romanowsky and H&E stained brain smears (Fig 3D and 3E). Critically, and in agreement with our TEM data, a single pRBC appeared sufficient to occlude a capillary. Consequently, uninfected erythrocytes and/or immature pRBCs could be observed in varying degrees of accumulation behind individual, mature pRBCs within, apparently obstructed, capillaries (Fig 3F–3K). We did not identify any cytoadherent pRBCs in venules or other larger calibre vessels. Conversely, we noted that a number of these larger vessels were distended and enriched with leukocytes, predominantly monocyte/macrophages, often dense with parasitic material (Fig 3L). We did not observe any extravascular pRBCs, though parenchymal macrophages (potentially microglial cells) enriched with parasitic material were observed occasionally (Fig 3M). These observations were validated in H&E stained sections of perfused brain tissue derived from *Pb* ANKA infected mice; whereby a number of microvessels were seen to be congested with erythrocytes, of which only a minority were parasitised (Fig 3N, 3NII, 3O & 3OIII). Critically, these results indicate that pRBC accumulation during ECM is dependent, or co-dependent, on parasite strain intrinsic capacities to deform within, and/or cytoadhere to, the cerebral microvasculature. Moreover, our results show that a single, pRBC appears sufficient to occlude and cause haemostasis within narrow murine cerebral capillaries. Critically, these observations imply that, despite the lower cerebral parasite biomass noted in ECM compared to HCM, the haemorheological consequences of pRBC accumulation may be similar in both.

### Intracerebral T-cell accumulation during *Pb* infections

**Fig 4:**
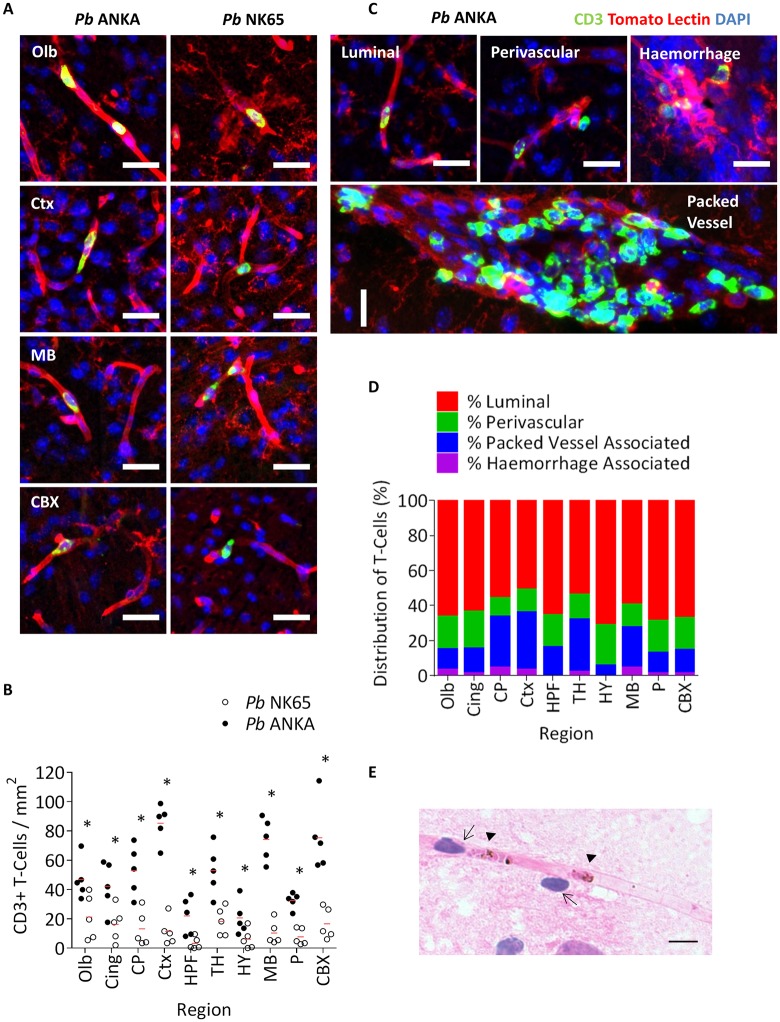
Intracerebral T-Cell burden is greater during ECM infection compared to non-ECM infection. C57BL/6 mice were infected with 1x10^4^ *Pb* ANKA GFP (n = 5) or 1x10^4^ *Pb* NK65 GFP (n = 5) pRBCs. All mice were euthanised on d7 p.i. when *Pb* ANKA infected mice developed late-stage ECM. Brains were removed from transcardially perfused mice and examined via immunofluorescence for the presence of CD3^+^ T-cells (green) in relation to lectin-labelled vasculature and activated macrophage (red), with nuclei counterstained blue. **(A)** Representative immunofluorescent images of vessel-associated CD3+ T-cells in the olfactory bulbs, cortex, midbrain and cerebellar cortex of brains from mice infected with *Pb* ANKA and *Pb* NK65. **(B)** Quantitation of CD3+ T-cells within the different brain regions of *Pb* ANKA and *Pb* NK65 infected mice. **(C)** Representative images demonstrating the different presentation of CD3^+^ T-cells in the brains of mice infected with *Pb* ANKA. **(D)** Quantification of the frequency (%) of CD3^+^ T-cells, (out of total observed CD3^+^ T-cells), that were; luminal, perivascular, haemorrhage-associated, or packed-vessel associated. **(E)** H&E stained brain smears taken from transcardially perfused mice on d7 p.i. (n = 5) showing luminal and abluminal lymphocytes (↑ note the distinctive dark nuclear staining and nuclear:cytoplasmic ratio) in close proximity to mature, sequestered pRBCs (▲). Dots represent mean of individual brains, with red lines mean of total brains. Bars represent mean frequency for all brains. Scale bars: A and C 25μm; E 5μm. *p ≤ 0.05 (unpaired t-test, no assumption made for consistent SD).

We next utilised our comparative model to perform a detailed quantitative examination of CD8^+^ T-cells. Whilst CD8^+^ T-cells are known to play a critical role in the development of ECM [27], we, and others, are still investigating their role in HCM. Due to an inability to utilise CD8 mAbs in fixed murine tissue [44], T-cells were labelled with CD3. Importantly, the majority of CD3^+^ T-cells in the brain are also CD8^+^ during ECM (S5 Fig). T-cells were observed in the cerebral vessels (identified by tomato lectin) of mice infected with either strain of *Pb*, and absent from the cerebral vessels of uninfected mice (Fig 4A, S6 Fig). Furthermore, whilst the number of T-cells was quantitatively greater in all brain regions from mice infected with *Pb* ANKA compared to *Pb* NK65, total T-cell numbers were low (Fig 4B). Indeed, despite ECM being a CD8^+^ T-cell dependent syndrome, T-cells were, on average, rarer than pRBCs in all corresponding brain regions from mice infected with *Pb* ANKA (Figs 2B and 4B). T-cells were predominantly found luminal or abluminal to the cerebral microvasculature, or as part of dense intravascular leukocyte accumulations in larger-calibre vessels during ECM (Fig 4C and 4D). Of note, the majority of leukocyte packed vessels contained lectin-labelled monocytes or macrophages, rather than CD3^+^ T-cells (S7 Fig). There was no evidence of extravasation of T-cells into the brain parenchyma, and the few T-cells observed within the parenchyma were associated with intracerebral haemorrhage (Fig 4C and 4D).

As pRBCs and/or parasite material were predominantly found in the same intracerebral compartments as T-cells (Figs 2C and 2D and 4C and 4D), we therefore hypothesised that the two might co-localise within the same subset of vessels. To test this hypothesis, we stained brain smears sampled from mice exhibiting fulminant ECM by H&E, and examined the association between morphologically-identified lymphocytes and parasite. We noted that while arrested pRBCs were often independent of lymphocytes, intracapillary or perivascular lymphocytes were invariably proximal to pRBCs (Fig 4E, S7 Fig). Our results indicate that, in the context of local pRBC accumulation, very few intracerebral T-cells are required for the development of ECM. Moreover, the co-localisation of T-cells with pRBCs implies that pRBCs may promote CD8^+^ T-cell accumulation.

### Intracerebral haemorrhage during *Pb* infections

**Fig 5:**
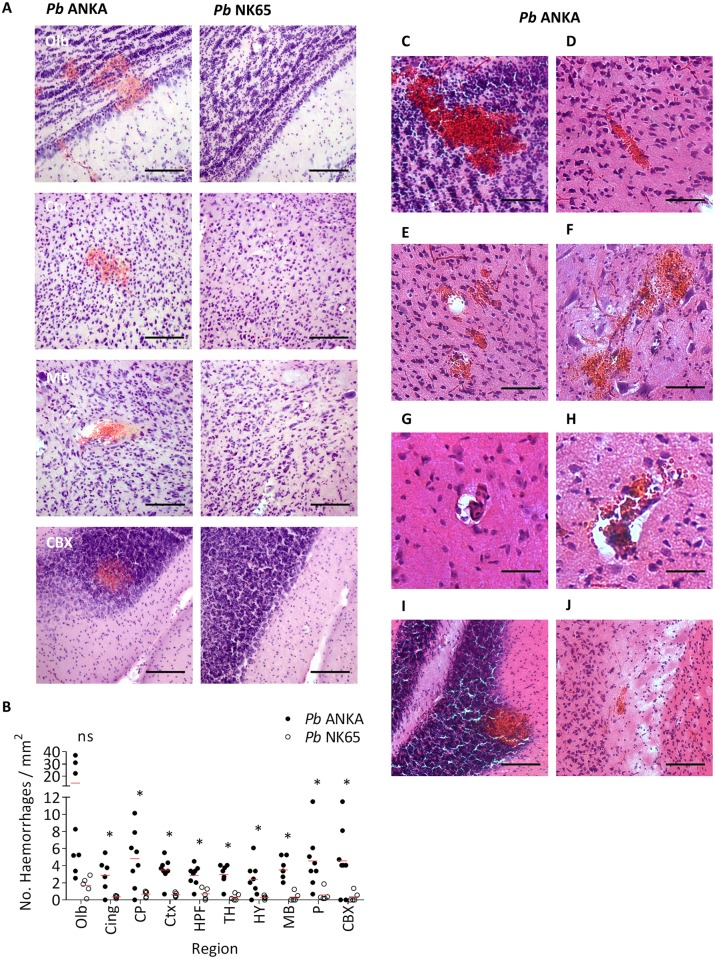
Incidence of intracerebral haemorrhage is greater during ECM infection compared to non-ECM infection. C57BL/6 mice were infected with 1x10^4^ *Pb* ANKA GFP (n = 8) or 1x10^4^ *Pb* NK65 GFP (n = 5) pRBCs. Mice were culled at d7 p.i. when *Pb* ANKA infected mice exhibited signs of late-stage ECM. Brains were removed from transcardially perfused mice and the nature and incidence of haemorrhage examined histologically via H&E staining. **(A)** Representative H&E images from the olfactory bulbs, cortex, midbrain and cerebellar cortex showing the relative severity and frequency of intracerebral haemorrhage in *Pb* ANKA and *Pb* NK65 infected mice. **(B)** Quantitation of haemorrhage within the different brain regions of *Pb* ANKA and *Pb* NK65 infected mice. The type and location of haemorrhage in the brains of mice experiencing ECM including: **(C)** extensive, amorphous haemorrhage from a large vessel in the olfactory bulbs; **(D)** extravasation of erythrocytes into the perivascular space of an occluded cortical microvessel; **(E)** several petechial haemorrhages in the cortex; **(F)** multiple haemorrhages at the denouements of a branching vessel; **(G)** thrombosed vessel with perivascular cuff; **(H)** thrombosed vessel with extravascular erythrocytes in the rarefied perivascular space and adjoining parenchyma; **(I)** large haemorrhage in the cerebellar grey matter (spanning both granular and Purkinje layers); **(J)** small haemorrhage in the external capsule white matter. Dots represent mean of individual brains, with red lines mean of total brains. Scale bars: A, I and J 75μm; C-F 50μm; G and H 25μm. *p ≤ 0.05 (unpaired t-test, no assumption made for consistent SD).

We next employed our comparative model to characterise the neurovascular-pathological events downstream of cerebral T-cell and pRBC accumulation during *Pb* ANKA infection. H&E staining demonstrated that intracerebral haemorrhage, a commonly described neuropathological feature in HCM and ECM, was quantitatively greater in all brain regions (with the notable exception of the olfactory bulbs) during *Pb* ANKA infection compared to *Pb* NK65 infection (Fig 5A and 5B, S8 Fig). Intracerebral haemorrhage was not observed in uninfected brains (S8 Fig). Large amorphous haemorrhages, perivascular bleeding and/or petechiae were all observed in the brains of mice during late-stage ECM (Fig 5C–5F). Whilst thrombosed vessels, with and without extravasated erythrocytes, were evident (Fig 5G and 5H), we saw no evidence of ring haemorrhages; a typical feature of HCM. As opposed to HCM, where haemorrhage occurs predominantly in the white matter [5], haemorrhaging was observed equivalently in the grey and white matter during ECM (Fig 5I and 5J). Our results, therefore, indicate that the frequency of haemorrhaging is increased during ECM, compared to uncomplicated malaria. However, the relative rarity of haemorrhage, in comparison to other pathological events, suggests it may not be the predominant cause of mortality during ECM.

### BBB permeability during *Pb* infections

**Fig 6:**
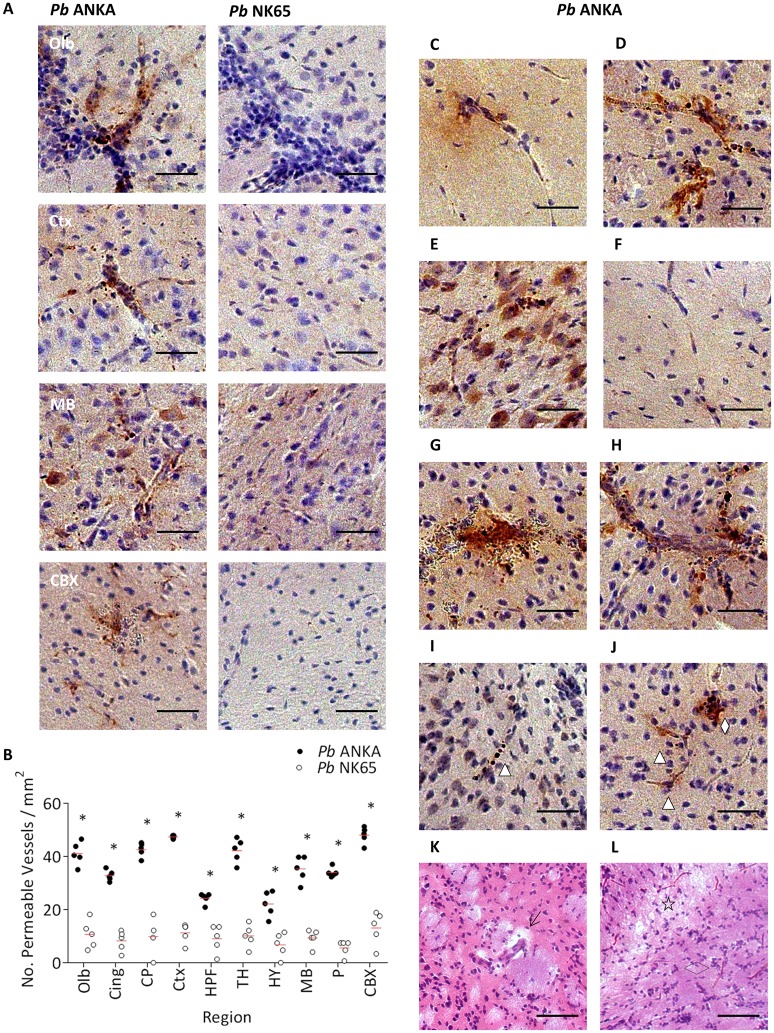
Cerebral oedema is more extensive during ECM infection compared to non-ECM infection. C57BL/6 mice were infected with 1x10^4^ *Pb* ANKA GFP (n = 5) or 1x10^4^ *Pb* NK65 GFP (n = 5) pRBCs. Mice were culled at d7 p.i. when *Pb* ANKA infected mice exhibited signs of late-stage ECM. Brains were removed from transcardially perfused mice and BBB disruption examined via IgG immunohistochemistry. **(A)** Representative images from the olfactory bulbs, cortex, midbrain and cerebellar cortex showing the relative prevalence of endogenous IgG in *Pb* ANKA and *Pb* NK65 infected mice. **(B)** Quantitation of permeable vessels within the different brain regions of *Pb* ANKA and *Pb* NK65 infected mice. Examples of vessels defined as permeable include: **(C)** vessel with a halo of IgGs; or **(D)** vessel associated with dense extravascular depositions of IgG and IgG immunoreactive astrocytes. **(E)** IgG immunoreactive neurons. **(F)** Occluded vessel with intravascular IgG. Patterns of IgG staining identified: **(G)** haemorrhage; **(H)** vessels packed with leukocytes; **(I)** microvessel with arrested pRBCs; and **(J)** permeable vessels without directly associated vascular pathological features (Δ), often, though, within the vicinity of vascular pathology (◊). Histological types of oedema visualised by H&E staining including: **(K)** microvessel with dilated perivascular space (↑) due to fluid accumulation; and **(L)** extensive parenchymal oedema resulting in vacuolation and/or rarefaction of the white matter (★) (compare to the compact fibre tract (◊) of non-oedematous white matter). Dots represent mean of individual brains, with red lines mean of total brains. Scale bar: 25μm. *p ≤ 0.05 (unpaired t-test, no assumption made for consistent SD).

Cerebral oedema resulting from enhanced BBB permeability is a common feature of HCM [5, 45, 46], and is thought to be a critical element of ECM pathogenesis [18, 26, 47]. However, to date, there has been no attempt to quantitatively assess the nature or presentation of vascular permeability during ECM. We assessed BBB permeability during *P*.*berghei* infection by staining for endogenous IgG, a serum protein ordinarily excluded from the cerebral parenchyma by an intact BBB [48]. We observed noticeably higher levels of IgG immunoreactivity, and, correspondingly, significantly increased numbers of permeable vessels, in the brains of mice infected with *Pb* ANKA compared to those infected with *Pb* NK65 (Fig 6A and 6B & S9 Fig). Positive IgG staining was not observed in uninfected brains (S10 Fig). Permeable vessels were characterised by a “halo” of IgG (Fig 6C), or dense extravascular depositions of IgG and/or IgG immunoreactive astrocytes (Fig 6D). In some brain regions (in particular the brainstem) from mice infected with *Pb* ANKA, but not *Pb* NK65, dense areas of IgG immunoreactive neurons were observed (Fig 6E). Although such neuronal staining has been defined as a historical marker of cerebral oedema [49, 50], as it was not possible to relate this parenchymal staining to particular blood vessels we did not quantify this pathological feature in our analysis. Furthermore, we excluded from our analysis any vessels that exclusively exhibited intravascular IgG staining (Fig 6F), as this identified occluded, not permeable, vessels. Extravasation of IgG was typically associated with haemorrhage (Fig 6G), larger calibre leukocyte-occluded vessels (Fig 6H), and/or intracapillary pRBCs (Fig 6I). Interestingly, scattered permeable vessels devoid of pRBCs, leukocytes or haemorrhage were also observed, however, these were typically in the vicinity of vessels exhibiting a specific pathological feature (as described above) (Fig 6J). Consistent with our IgG staining, we also observed clear histological evidence of cerebral oedema during ECM (Fig 6K and 6L), suggesting the severity and/or prevalence leakage must be substantial. Combined, these results, in agreement with the literature [26, 51], show that BBB permeability is widespread within the brain during ECM, and that vascular leakage and subsequent oedema is significantly greater during *Pb* ANKA than during *Pb* NK65 infection. Importantly, our data also shows that whilst BBB permeability during ECM is typically associated with parasitised microvessels, intravascular accumulations of leukocytes or haemorrhage, permeable vessels devoid of any such associated vascular pathology are also present. Whilst this could indicate that the pathological event triggering the vascular leakage cleared subsequent to analysis, it may also suggest that soluble mediators expressed by the vascular bed at distinct pathological sites may induce diffuse BBB permeability during ECM.

### Apoptosis during *Pb* infections

**Fig 7:**
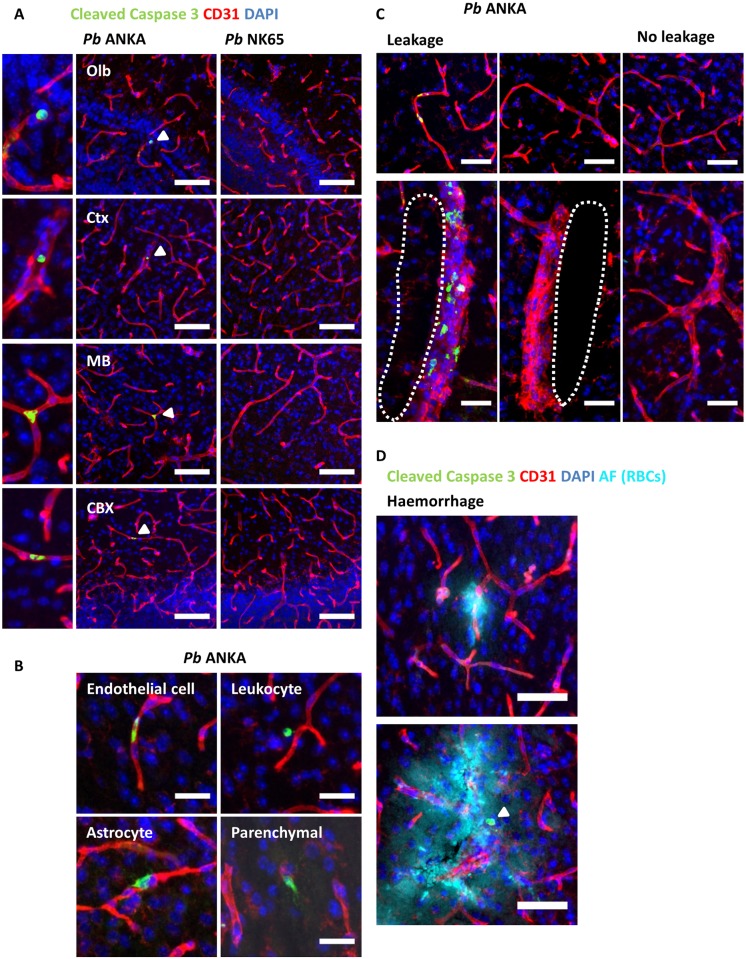
Vascular leakage occurs independently of endothelial cell apoptosis. C57BL/6 mice were infected with 1x10^4^ *Pb* ANKA GFP (n = 4) or 1x10^4^ *Pb* NK65 GFP (n = 4) pRBCs. Mice were culled at d7 p.i. when *Pb* ANKA infected mice exhibited signs of late-stage ECM. Brains were removed from transacardially perfused mice and examined via immunofluorescence for the presence of cleaved Caspase 3 (Green) in relation to lectin-labelled vasculature and macrophages (red), with nuclei counterstained blue. **(A)** Representative immunofluorescent images demonstrating the degree of cellular apoptosis in the olfactory bulbs, cortex, midbrain and cerebellar cortex of brains from mice infected with *Pb* ANKA and *Pb* NK65, CC3^+^ cells denoted by (Δ) in *Pb* ANKA infected mice shown in magnified panel (left). **(B)** Representative images showing the types of CC3^+^ cells observed in the brains of mice infected with *Pb* ANKA **(C)** Vessels with and without CC3^+^ endothelial cells can be seen adjacent to oedema, identified by uncondensed parenchyma and/or perivascular dilation (delineated by dotted line). Non-odematous tissue with compact parenchyma and indiscernible perivascular spaces are in the right panels for comparison. **(D)** Haemorrhage, identified by auto-fluorescent RBCs (teal), can be seen proximal to disrupted CD31 staining (top panel) and occasional extravascular CC3^+^ cells (Δ) (bottom panel). Scale bars: A 75μm; B 25μm; C-D 50μm.

We next examined the mechanistic basis for intracerebral vascular permeability during ECM. In particular, as it has been proposed that CD8^+^ T-cells promote cytolysis of cross-presenting endothelial cells [30, 31], we assessed the level of cellular apoptosis in the brains of mice infected *Pb* ANKA or *Pb* NK65. Cellular apoptosis (detected by expression of cleaved Caspase 3 (CC3)) was rarely observed in the brains of mice infected with *Pb* ANKA, was even less frequent during *Pb* NK65 infection, and was not observed in the brains of uninfected mice (Fig 7A & S11 Fig). The majority of the apoptotic events during ECM were associated with the vasculature, and were predominantly endothelial cells, leukocytes, or, more infrequently, astrocytes (Fig 7B). Atypical parenchymal cellular apoptosis did not appear to be neuronal, based on morphological criteria (Fig 7B). Notably, cerebral oedema (characterised by uncondensed parenchyma and/or perivascular dilation) was observed proximal to vessels with and without evidence of apoptotic ECs (Fig 7C), suggesting leakage is not dependent on EC loss. Haemorrhages were associated with disrupted vessel staining, but not EC apoptosis; indicating non-apoptotic mechanisms are likely responsible for haemorrhage (Fig 7D). We saw no evidence of conterminous vascular degeneration adjacent to haemorrhage, i.e. endothelial cell apoptosis within the afflicted vascular bed; though we did observe apoptotic leukocytes focal to the lesion (Fig 7D). Importantly, the area and number of vessels was unaltered during *Pb* ANKA and *Pb* NK65 infection (S12 Fig). This implies that vascular loss is a limited and stochastic event associated exclusively with haemorrhage, and not a central contributor to the cerebral oedema seen during ECM. Combined, these results indicate that programmed cell death of ECs is highly unlikely to be the major mechanism provoking the widespread vascular leakage that occurs during ECM.

### Alternative mechanisms of leakage during *Pb* infection

**Fig 8:**
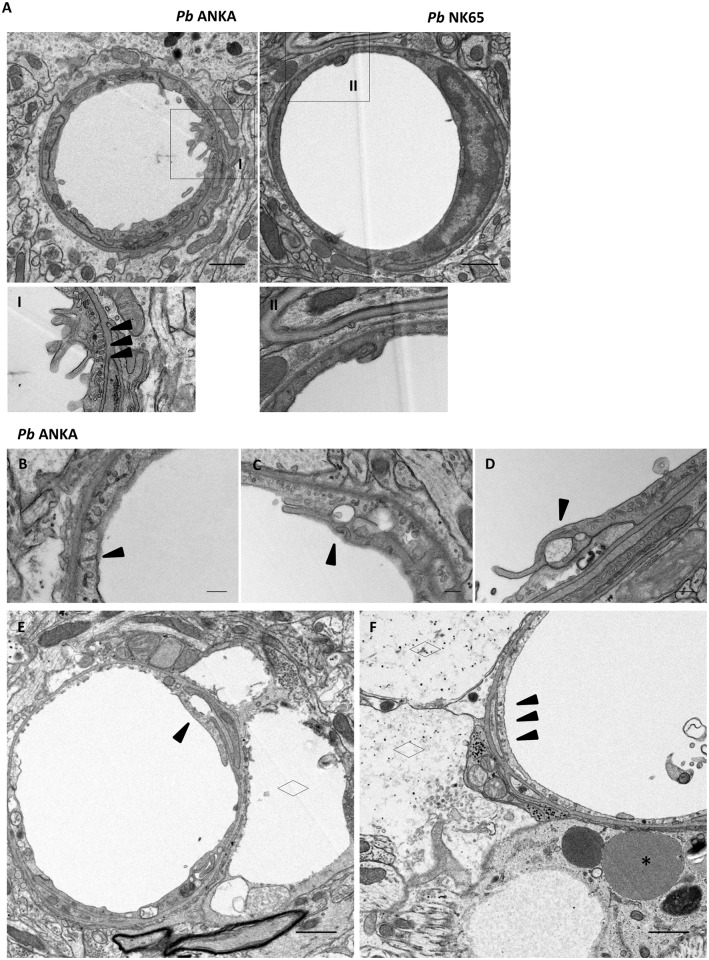
Vascular leakage is associated with dysregulation of transcellular and paracellular transport mechanisms during ECM. C57BL/6 mice were infected with 1x10^4^ *Pb* ANKA GFP (n = 3) or 1x10^4^ *Pb* NK65 GFP (n = 3) pRBCs. Mice were culled at d7 p.i when *Pb* ANKA infected mice exhibited signs of late-stage ECM. Brains were removed from transcardially perfused mice and processed for TEM **(A)** Electron micrographs of representative vessels from *Pb* ANKA and *Pb* NK65 infected mice. Note the thickened endothelium and narrowed lumen consistent with vasospasm, cytoplasmic extensions and extensive caveolae seen during *Pb* ANKA infection. **(i and ii)** Digitally magnified panels showing endothelial cytoplasmic extensions and extensive caveolae (▲) specifically during *Pb* ANKA infection. The potential mechanisms of microvessel leakage during *Pb* ANKA infection include: **(B)** transendothelial channel (▲); **(C)** vacuolar cleft (▲) associated with an endothelial cell tight junction, numerous caveolae adjacent; **(D)** cleft (▲) in an atypical endothelial cell tight junction; **(E)** cleft within microvascular tight junction (▲) adjacent to fluid accumulation within the perivascular space (◊); **(F)** caveolae (▲) proximal to astrocyte end-feet swelling (◊), extravasated RBCs (*) can also be visualised. Scale bars: A, E & F 1μm; B-D 2μm.

As widespread BBB disruption during ECM did not appear to be associated with a loss of cerebral ECs, we subsequently sought to examine whether alterations in transcellular and/or paracellular transport mechanisms could account for enhanced brain vessel permeability. Utilising TEM, we observed extensive pseudopodia, or cytoplasmic extensions, in a number of vessels during ECM. Pseudopodia were seen only rarely on cerebral ECs during *Pb* NK65 infection and not seen in uninfected samples (Fig 8A & S13 Fig). Caveolae were abundant in cerebral ECs in the brains of mice infected with *Pb* ANKA compared to *Pb* NK65 (Fig 8AI and 8AII). In some vessels aggregations of caveolae appeared to form transendothelial pores specifically during ECM (Fig 8B). In addition, large clefts in the cerebral microvascular tight junctions were observed during ECM, but were only rarely seen in uncomplicated malaria infection (Fig 8C and 8D). Interestingly, clefts in tight junctions and accumulations of caveolae were observed proximal to cerebral oedema (Fig 8E and 8F). This suggests that alterations to the transcellular and/or paracellular permeability of the brain microvasculature may be responsible for the vascular leakage observed during ECM.

Additionally, we noted an apparent thickening of the basement membrane and luminal contraction consistent with vasospasm in a number of the cerebral microvessels of mice infected with *Pb* ANKA compared to *Pb* NK65 (Fig 8A). However, due to the natural range of capillary diameter within the rodent brain [52], definitive assessment of vasospasm was not possible in our analysis.

### Myelin and axonal injury during *Pb* infection

**Fig 9:**
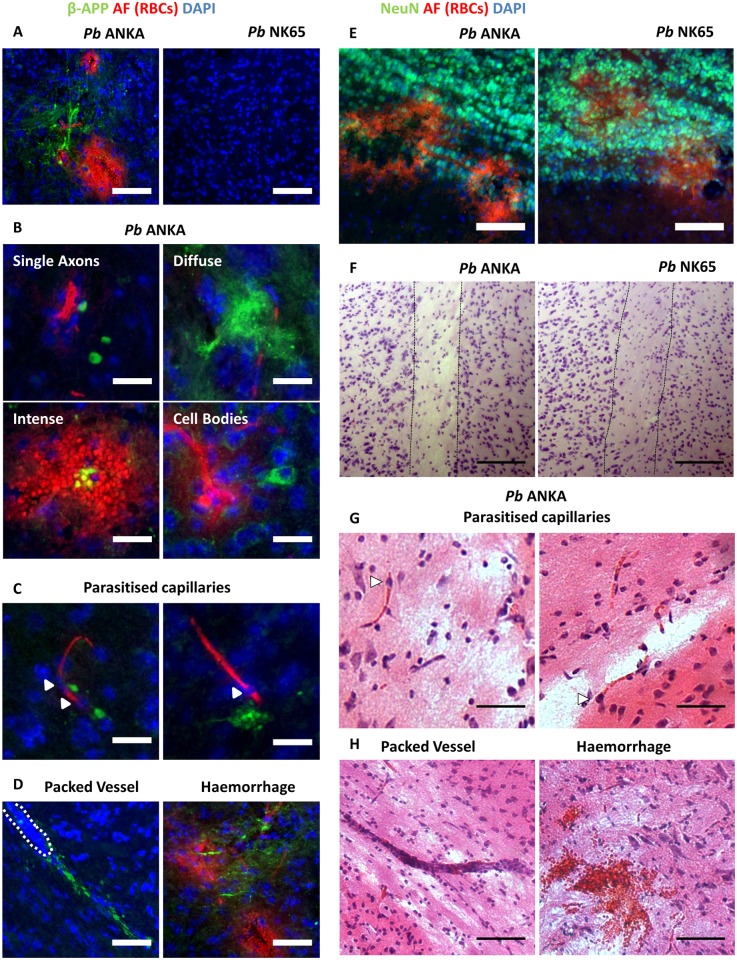
ECM is associated with demyelination and axonal dysfunction. C57BL/6 mice were infected with 1x10^4^ *Pb* ANKA GFP (n = 7) or 1x10^4^ *Pb* NK65 GFP (n = 5) pRBCs. Mice were culled at d7 p.i. when *Pb* ANKA infected mice exhibited signs of late-stage ECM. Brains were removed from transcardially perfused mice and processed for immunofluorescent and histological examination. **(A)** Representative images show presence and absence, respectively, of β-APP (green) accumulation within white matter tracts (Pons) during *Pb* ANKA and *Pb* NK65 infection. Cell nuclei are blue and auto-fluorescent (AF) RBCs red. **(B)** Patterns of β-APP staining seen during ECM. β-APP accumulation adjacent to: **(C)** erythrocyte congested capillaries, DAPI labelled erythrocytes are likely pRBCs (Δ); **(D)** leukocyte-packed vessel (denoted by dashed line), and haemorrhage. **(E)** Disruption of neuronal architecture during *Pb* ANKA and *Pb* NK65 infection in the olfactory bulbs. NeuN+ neurons (green), auto-fluorescent (AF) RBCs red and cell nuclei blue. **(F)** Extensive white matter disruption visualised by H&E staining during *Pb* ANKA, but not *Pb* NK65, infection (dotted black lines demarcate boundaries of the white matter (external capsule)). Myelin pallor, fragmentation and/or gross demyelination seen adjacent to **(G)** erythrocyte-congested capillaries (Δ), **(H)** leukocyte packed vessel and haemorrhage. Scale bars: A, D, E & F 75μm; B, C & G 25μm and H 50μm.

Whilst it is not entirely clear how the cerebral vascular pathology that characterises HCM influences parenchymal brain function to induce coma and death, it has been shown that axonal injury (AI) and myelin loss are common neuropathological features of the syndrome [5, 13]. Conversely, there is currently no histopathological data defining the neurological abnormalities that occur during ECM. Using our comparative model, we observed significant evidence of AI specifically during ECM, as shown by β-APP, a protein that accumulates at sites of AI (Fig 9A). Several patterns of β-APP staining were evident: labelling of single axons; diffuse regions; more intense regions; and scattered, intensely-immunoreactive neuronal cell bodies (Fig 9B). AI was noted adjacent to specific vascular pathological features during ECM, including: parasitised capillaries; leukocyte-packed vessels; and haemorrhage (Fig 9C and 9D). In contrast, we observed that the neuronal architecture (defined by NeuN staining) was broadly unaltered during both *Pb* ANKA and *Pb* NK65 infection (S14 Fig). Moreover, and consistent with our data in Fig 7, we saw no evidence of apoptotic neurons in the brains of *Pb* ANKA or *Pb* NK65 infected mice (S14 Fig). However, we did note neuronal lesions proximal to some haemorrhages in the olfactory bulbs of mice infected with both *Pb* ANKA and *Pb* NK65 (Fig 9E). In addition to AI, there was evidence of extensive myelin pathology specifically in the brains of mice infected with *Pb* ANKA (Fig 9F). We observed discrete regions of gross demyelination, and myelin pallor and fragmentation associated with parasitised capillaries, leukocyte-packed vessels and haemorrhage (Fig 9G and 9H). Collectively, these results suggest the nature of cerebral parenchymal damage is comparable in HCM and ECM, and provide a logical explanation for the clinical similarity in the transient and long-term neurological dysfunction that occurs during and post HCM and ECM.

### Correlation between different histopathological parameters during *Pb* ANKA infection

**Fig 10:**
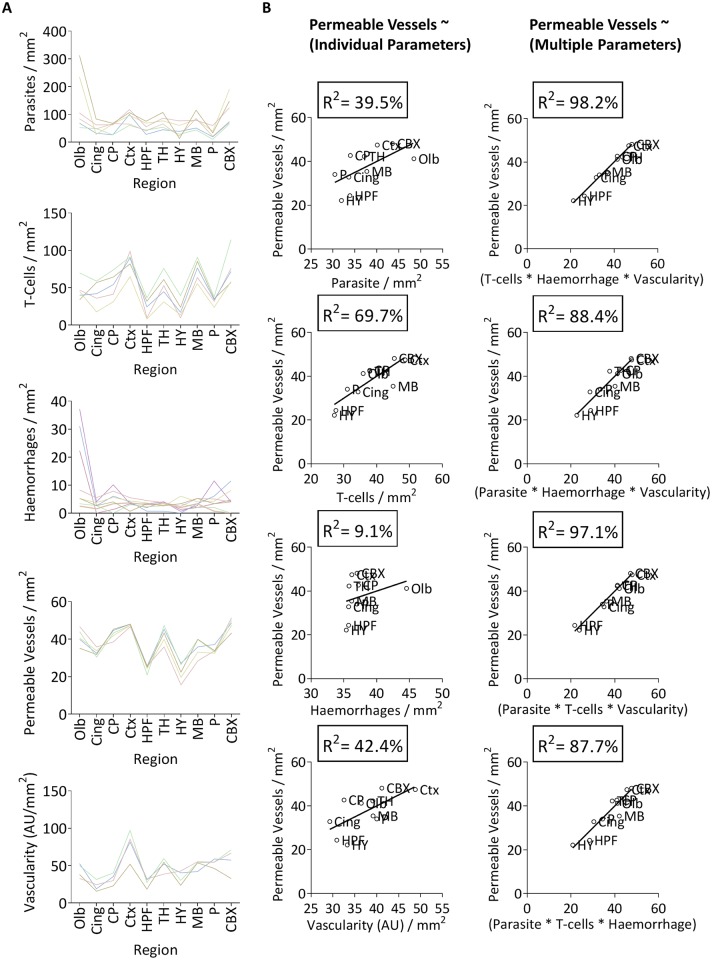
The deterministic and multifactorial nature of ECM. **(A)** The magnitude of the various pathogenic features within all ten brain regions, plotted separately for individual mice with late stage ECM (n = 4–8). **(B)** Generalised linear model analyses assessing the contribution of the pathogenic features, in isolation and in combination, in promoting vascular leakage within the brain during late-stage ECM. Each data point represents the mean of the group for each region during late-stage ECM (N = 4–8).

To further improve our understanding of the pathogenesis of ECM, we examined the spatial nature of the defined pathological features within individual ECM-affected brains. Although the magnitude of parasite accumulation varied between cases of ECM, we observed broad trends in regional parasite accumulation within individual cases of ECM (Fig 10A), with parasite load typically greater in the Olb, Ctx, TH, MB and CBX than the other regions (Fig 10A). The number of haemorrhages was typically highest in the Olb (dramatically in some cases), but was of low level and variable in other regions between brains (Fig 10A). Conversely, the spatial nature of T-cell accumulation and permeable vessels was highly consistent in all cases of ECM, with the pathological features showing strong regional overlap (Fig 10A). Thus, although ECM is clearly a graded syndrome where the magnitude of pathological events varies from cases to case, the pathology is not stochastic with specific brain regions consistently more severely affected than others. This implies that architectural or physiological properties may predispose specific brain regions to malaria-induced cerebral pathology.

Given the conserved and equivalent regionalisation, we examined the co-dependent relationship(s) between the identified pathological processes and their relative (individually and in combination) contribution in promoting ECM. As expected, there was a significant correlation between regional parasite and T-cell load, which is in agreement with the observation that parasites and T-cells co-localise within the tissue (S15 Fig & Fig 4E). Interestingly, the degree of vascularity did not correlate significantly with the number of parasites, haemorrhages or permeable vessels within brain regions, suggesting that vessel quality, rather than quantity, is more critical in determining regional pathological burden during ECM (S15 Fig). Importantly, through using generalised linear modelling, we found that combinations of histopathological parameters were, generally, better predictors of vascular permeability (which our results indicate is the major pathological event during ECM) within a brain region, compared to any single histopathological parameter (Fig 10B & S1 Table). For example, parasite burden, T-cell load and degree of vascularity combined were a better predictor of the number of permeable vessels within a brain region, than any of these factors in isolation (Fig 10B). Collectively, these data support the assertion that ECM is a multifactorial neuropathology that does not develop in response to a singular, dominant pathological event within any region of the brain. However, parasite accumulation within the brain appears to be a proximal event important for intracerebral T-cell accumulation, localisation and function, which ultimately provokes vascular dysfunction.

## Discussion

In this study we have utilised detailed histopathological investigations, analogous to those used in the study of HCM, to definitively assess the relative merit of the ECM model for the study of HCM. Critically, by contrasting cerebral pathology observed during ECM with that during uncomplicated malaria infection, we have also substantially resolved the specific intracerebral events associated with the development of the ECM syndrome.

We demonstrated that the global accumulation of pRBCs within the capillaries of the murine brain is a specific and cardinal feature of ECM. The compartmentalisation of pRBCs predominantly within the cerebral microvasculature during ECM, rather than pooled within haemorrhage or entrapped by intravascular leukocyte aggregations, indicates that intracerebral pRBCs likely play a causal role in the late-stages of the murine syndrome. Indeed, the comparable efficacy of anti-malarial drug treatment in reversing ECM and HCM strongly implies that intracerebral pRBCs play an active role in the late-stages of both mouse and human malaria-induced cerebral pathology [2, 22]. However, our results also highlight clear differences in the presentation and magnitude of parasite accumulation during ECM compared with HCM. We found that intracerebral pRBCs were typically observed individually and irregularly distributed within brain capillaries during ECM. In contrast, a number of studies have shown that *Pf* parasitised erythrocytes are densely packed and congest significant lengths of the microvasculature during HCM [8–11, 43, 53]. Nevertheless, despite these differences, our results imply that some of the consequences of intracerebral pRBC accumulation may be the same in mice and humans.

**Fig 11:**
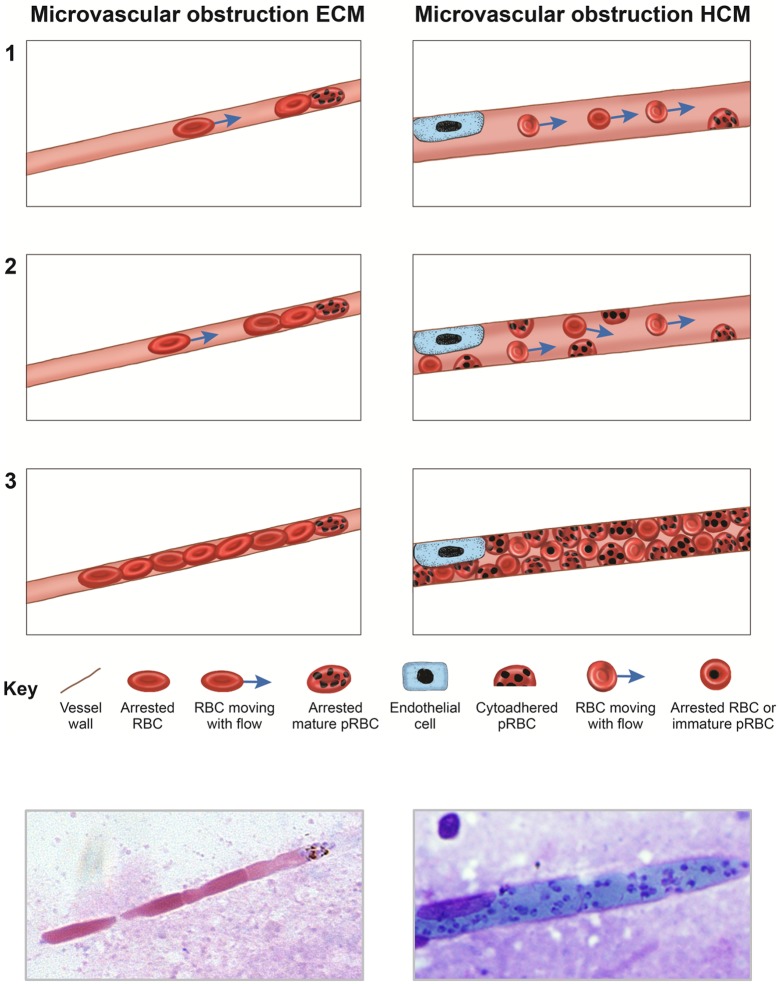
Models for pRBC-dependent microvascular obstruction in ECM and HCM. In a step-wise fashion **(1–3)**, models show how pRBC-dependent microvascular obstruction occurs during malaria-induced encephalopathy in mice (left panels) and humans (right panels), respectively. Bottom left panel is a quik-diff stained brain smear taken from a transcardially-perfused mouse during the late-stages of ECM; uninfected erythrocytes can be seen to accumulate behind an arrested late-stage schizont within a narrow murine capillary (reproduced from Fig 3H). Bottom right panel is a reverse Fields stained brain smear taken from a patient who died from Cerebral Malaria; the length of the distended capillary is densely packed with mature trophozoites (courtesy of Prof. D Milner).

We have shown that in ECM, as in HCM, pRBC-dependent occlusion of brain capillaries and haemostasis are associated features of disease. In ECM, the width of murine cerebral capillaries necessitates the single-file passage of extensively deformed tubular erythrocytes. Consequently, the arrest of a single pRBC appears to be sufficient to occlude a murine brain capillary, and thus cause localised haemostasis. Conversely, in HCM, the available histopathological evidence suggests that mechanical obstruction of brain capillaries and resultant haemostasis depends on the incremental accumulation of large numbers of cytoadherent pRBCs [8–11, 43, 53] (Fig 11). Indeed, a process whereby uninfected erythrocytes are initially able to squeeze past cytoadhered pRBCs, until a critical threshold of pRBCs is reached within a vessel and mechanical obstruction occurs, is the only logical explanation for the high intracerebral pRBC sequestration indexes observed in HCM (i.e. in one study it was shown that that, on average, 66.5% of intracerebral RBCs were parasitised, compared to 1.4% in the peripheral blood [42]). Thus, although the presentation of pRBC accumulation in the cerebrovasculature may be different in ECM and HCM, our results provide a rational potential explanation for the comparable alterations in blood flow observed *in vivo* during murine and human malaria-induced cerebral pathology [54–59]. Whether the differential natures of pRBC-mediated vascular occlusion during ECM and HCM depend upon established differences in murine and human capillary diameter (average 3um vs 6.4um) [60–62], or upon the degree to which murine and human cerebral capillaries can mechanically dilate, requires further investigation.

The major question, therefore, is how do *Pb* ANKA parasites accumulate within the brain during ECM? Whilst we did not observe any knob formation on *Pb* infected erythrocytes (and thus no knob-based cytoadhesion as is observed with *Pf* [9, 11, 63, 64]), we did observe the occasional electron dense spot on the surface of some parasitised erythrocytes adjacent to the cerebral vasculature during ECM. Such events appear comparable to the knob-independent forms of sequestration demonstrated by *Pf* infected RBCs *in vitro* [65], and may reflect the *in vitro* capacity of *Pb* ANKA infected RBCs to bind VCAM-1 expressed by brain ECs [66]. However, electron dense spots were not observed consistently on pRBCs within perfused brain microvessels, implying other mechanisms must also contribute to intracapillary pRBC accumulation during ECM. The equivalent propensity of *Pb* ANKA and *Pb* NK65 to parasitise larger reticulocytes suggests pRBC size does not determine the capability of different *Pb* strains to immobilise within cerebral capillaries [67]. However, it may be that some strain intrinsic qualities relating to the rheological properties of pRBCs, including deformability, specify the intracerebrovascular accumulation capacity of *Pb* infected erythrocytes. Indeed, *Pasini et al* have previously demonstrated differences in the repertoire of proteins expressed by ECM-inducing and non-ECM inducing *Pb* strains [68]. Crucially, although the differential expression of these proteins did not directly alter the cytoadherent capabilities of the parasites, it was not assessed whether the repertoire of protein expression influenced pRBC rheology. However, we also noted, consistent with a previous study [55], that vasospasm appeared to be a specific feature of *Pb* ANKA infection. A narrowing of the vascular lumen would exacerbate any rheological impairment, suggesting variant host responses to different *Pb* strains may also contribute to haemostasis during ECM.

The conclusion that mature *Pb* ANKA infected RBCs become mechanically trapped within narrow murine brain capillaries during ECM, rather than accumulating as a result of strong cytoadherence, is supported by our failure to detect independent pRBC accumulation in venules or other large calibre vessels. Indeed, we previously failed to observe long-lasting pRBC adherence within the wider pial vessels using intravital microscopy [26]. Moreover, as opposed to observations in HCM [69], we occasionally observed extravascular pRBCs in the perivascular spaces and within haemorrhages (an observation that was relatively more common in the meninges [26]), suggesting *Pb* ANKA pRBCs are not tightly adhered to the brain endothelium and thus freely liberated from vessels upon necrotic EC loss. Further work will be required to identify the precise factors that dictate the capacity of specific *Pb* strains to accumulate within the cerebrovasculature during infection to cause ECM. Nevertheless, although our results suggest that ECM is a good model to understand the downstream effects of intracerebral pRBC accumulation and resultant haemostasis, they also imply that it is not a good system to investigate the consequences of direct pRBC cytoadherence to brain ECs. However, relevantly, the importance of direct (parasite sequestration-dependent) compared with indirect (inflammation-driven) activation and dysfunction of human brain ECs in the development of HCM is yet to be definitively identified [70].

Although our data indicates that pRBC-mediated occlusion of the cerebrovasculature appears to occur during ECM, as is observed during HCM, microvascular obstruction alone cannot explain the full repertoire and nature of murine and human malaria-induced cerebral pathology [71]. Beyond ischemia, which does not satisfactorily explain our neuropathological findings, nor the rapidly reversible nature of ECM and HCM, it is unclear how parasitised erythrocytes and subsequent microrheological alterations promote coma and death. Murine studies propose the cross-presentation of merozoite-derived parasitic material by cerebral ECs licences cerebrovascular CD8^+^ T-cells to promote vascular leakage during ECM [30, 31]. Accordingly, we observed significantly greater numbers of intracerebral CD8^+^ T-cells during *Pb* ANKA infection compared to *Pb* NK65 infection. CD8^+^ T-cells were located within the intra- or perivascular space and, interestingly, were typically proximal to pRBCs or parasite material during ECM. Indeed, there was a strong correlation between parasite and T-cell load within the different brain regions. Thus, our data, in the context of the current literature, suggests intracerebral pRBC accumulation and subsequent microvascular obstruction fulfils three roles vital to the pathogenesis underlying ECM: 1) to provide parasite antigen for cross-presentation; 2) to promote haemostasis, thus ensuring the necessary microenvironment in which cerebral ECs are able to obtain merozoites for cross-presentation, which, under physiological flow conditions, would normally be rapidly cleared; and 3) to instigate signals important for the cerebrovascular localisation of CD8^+^ T-cells. Notably, despite the importance of CD8^+^ T-cells in promoting ECM [27], they were observed relatively rarely within the brains of mice infected with *Pb* ANKA; being less frequent than pRBCs and substantially less populous than macrophages and monocytes. The reasons for the differences in intracerebral macrophage/monocyte and CD8^+^ T-cell numbers, considering they are similar located in the cerebrovasculature and likely depend on the same EC-derived ligands/integrins, are not clear, but may depend upon temporal differences in recruitment [72]. Nevertheless, the general rarity of CD8^+^ T-cells within the brain implies that very few are required to promote ECM and, furthermore, may explain the failure, thus far, to consistently locate this cell population in HCM histopathological studies [5, 9].

CD8^+^ T-cell-dependent vascular leakage is currently considered critical to the development of ECM [26, 51]. Consistent with this, we observed greater evidence of cerebral oedema during *Pb* ANKA infection compared to *Pb* NK65 infection. The causes of oedema during ECM appeared multifactorial in origin as, in accordance with HCM histopathological study [5], we detected increased permeability around haemorrhages, parasitised capillaries, intravascular leukocyte accumulations, and some scattered microvessels devoid of any specific vascular-pathological feature. Indeed, we observed significant correlation between the number of permeable vessels within a brain region and parasite, T-cell or haemorrhage load. These observations additionally support the hypothesis that extensive cerebral oedema may promote the fatal cerebral swelling that occurs during human and murine malaria-induced encephalopathy [25, 26, 51, 73, 74].

**Fig 12:**
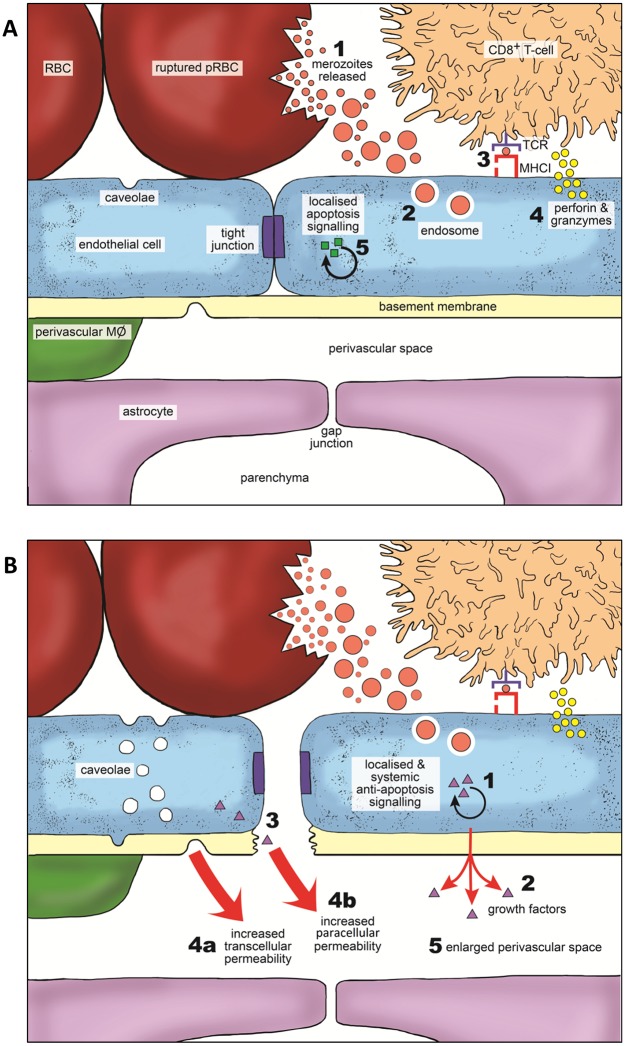
Model for ECM-associated vascular leakage. **(A)** 1) Arrested Schizont ruptures in the cerebral capillary releasing merozoites. 2) pRBC-dependent alterations in cerebral capillary blood flow facilitate EC phagocytosis of merozoites and digestive vacuoles. 3) ECs cross-present parasite antigen on MHC class I molecules to CD8^+^ T-cells. 4) CD8^+^ T-cells degranulate and release cytotoxic perforin and granzyme molecules). 5) The cytolytic molecules induce apoptosis signalling within the target EC. **(B)** 1) Target EC induces anti-apoptosis signalling pathways to counteract cytolysis via 2) the secretion of growth factors. Growth factors act in an autocrine fashion on the target EC and 3) paracrine fashion on neighbouring ECs. This results in: 4a) the upregulation of caveolae within affected ECs; and 4b) alterations to intercellular EC tight junctions. 5) The enhanced permeability of the cerebrovasculature leads to enlargement of the perivascular spaces and formation of oedematous parenchymal tissue, which precipitate fatal increases in intracranial pressure.

Our data also provides significant information on the mechanism through which CD8^+^ T-cells cause vascular leakage during ECM. We showed that, whilst haemorrhage was evidently secondary to EC damage, the majority of vascular leakage occurred independently of EC loss or apoptosis. This suggests, consistent with our previous study [26], and despite the importance of CD8^+^ T-cell cytolytic functions in promoting BBB dysfunction during ECM [28, 29], that vascular leakage occurs without EC loss during murine malaria-induced cerebral pathology. Instead of vascular loss, we demonstrated that significant vascular remodelling occurs specifically during ECM. Clefts within the microvascular tight junctions and increased levels of caveolae were noted within ECs adjacent to cerebral oedema. Moreover, pronounced cytoplasmic extensions, or pseudopodia, were evident in several brain vessels. Such alterations in EC morphology are traditionally viewed as hallmarks of angiogenesis [75]. Consequently, our observations support a model whereby CD8^+^ T-cells, via Granzyme B and perforin [28, 29], promote a vascular stress response in ECs, resulting in the production of angiogenic factors which, although protective against cellular apoptosis, cause lethal alterations to the paracellular and/or transcellular permeability of the cerebrovasculature during ECM (Fig 12). Supporting this hypothesis, in a variation of Theiler’s murine encephalomyelitis model of MS, CD8^+^ T-cells have been shown to promote the production of VEGF in a perforin-dependent fashion, causing vascular leakage without cerebral EC loss [76]. Importantly, this scenario affords a rational explanation as to why rapid recovery from ECM and restoration of vascular integrity can occur after anti-malarial drug treatment, which would not be possible if vascular leakage were determined by extensive and irreversible EC loss. Intriguingly, not only has vascular leakage in the absence of EC loss been observed in HCM histopathological studies [5], but so has disruption of intercellular tight junctions [12, 77]. Our data, in conjunction with the literature, imply the mechanisms underlying vascular dysfunction may be conserved between humans and mice during malaria-induced cerebral pathology. These observations further underline the requirement for highly resolved histopathological studies to be performed to specifically examine the potential importance of CD8^+^ T cells in the pathogenesis of HCM.

Therefore, the final and critical questions are 1) how does the neurovascular pathology characterising ECM influence parenchymal brain function to induce coma and death, and 2) are the pathways to neurological dysfunction conserved between ECM and HCM? We demonstrated that axonal injury (AI) is a significant and specific feature of ECM, as evidenced by positive β-APP staining, a protein that accumulates at sites axonal damage [78]. Axons extend significant distances, and are thus dependent on a huge number of microvessels for the provision of oxygen and glucose to permit their metabolically expensive functions [13]. Indeed, AI has been shown to occur in response to both hypoxia and hypoglycaemia [79, 80]. Accordingly, we observed AI proximal to erythrocyte congested vessels, suggesting microvascular obstruction likely accounts for much of the AI observed during ECM. However, we also observed AI adjacent to haemorrhage and intravascular leukocyte accumulation, indicating that axonal dysfunction is potentially a common mechanism through which multiple pathological parameters of ECM impair neurological function. Interestingly, β-APP accumulation within axonal tracts may represent reversible axonal damage, consistent with the rapid neurological recovery observed in ECM after anti-malarial drug treatment [81]. In addition to AI, we frequently observed myelin damage during ECM, with areas of myelin pallor and vacuolation seen proximal to erythrocyte-congested vessels, and physical loss of the myelin sheath detected adjacent to haemorrhage. The progressive accumulation of myelin and axonal damage would explain the graded and sequential neurological dysfunction observed during ECM, including ataxia, fitting and reduced responsivity [82]. Coma may ensue due to a culmination of axonal and myelin pathology (potentially as a programmed neuroprotective response to lower cerebral metabolic demand). Importantly, the nature of AI and myelin damage observed in ECM is highly similar to that reported in HCM [5, 13]. Moreover, AI and myelin damage are colocalised to specific, shared vascular-pathological features, including haemorrhage and pRBC occluded microvessels, in both ECM and HCM [5, 13] Combined, this implies that the mechanisms responsible for the reversible and/or permanent neurological dysfunction observed during and after an episode of malaria-induced encephalopathy, are very likely conserved between mice and humans.

In summary, the results in this manuscript show, in significant detail and for the first time, that mature pRBCs specifically accumulate within the cerebral capillaries during ECM. Although the mechanism and presentation of pRBC accumulation during ECM is significantly different to that observed during HCM, there appears to be overlap in the pathological impact of parasite-induced haemostasis during both syndromes. It is, however, clear that ECM does not appear to be a good model to study the impact and role of pRBC cytoadherence to cerebral blood vessels in the development of malaria-induced cerebral pathology. Nevertheless, in spite of this, a number of pathological features of HCM are observable and appear comparable in nature during ECM, including; BBB disruption, AI and myelin damage. The critical role of CD8^+^ T cells in initiating the ECM syndrome, when roles for the cells have yet to be revealed in HCM, remains a divisive point. Based upon our data, showing significant similarities in vascular and parenchymal pathology in ECM and HCM, and the fact that so few cerebrovascular CD8^+^ T cells can dominantly drive cerebral pathology during ECM, detailed investigations of the role of CD8^+^ T cells during HCM are warranted. Nonetheless, if, after detailed investigation, it is ultimately found that CD8^+^ T cells cannot be involved in the development and progression of HCM, the observation that many of the pathological features of ECM are similarly found in HCM indicates that convergent signals may ultimately drive the same severe pathological manifestations during ECM and HCM. Thus, when used appropriately (i.e. not solely relying on KO mouse studies to examine pathogenesis or employing treatments before ECM develops), our collective results support the utilisation of the ECM model to understand the pathological events secondary to pRBC accumulation in HCM. In addition, careful utilisation of the ECM model may be useful for the identification of novel adjunct therapies for the repair and resolution of the vascular and parenchymal damage that occurs similarly within the established ECM and HCM syndromes.

## Materials and methods

### Ethics

All animal work was approved following local ethical review by the University of Manchester Animal Procedures and Ethics Committees and was performed in strict accordance with the U. K Home Office Animals (Scientific Procedures) Act 1986 (approved H.O. Project License 70/7293).

### Mice and infections

Female and male 8–10 week old C57BL/6 mice were purchased from Charles River and maintained in individually ventilated cages at the University of Manchester. Cryopreserved *P*.*berghei* ANKA GFP [83] and *P*.*berghei* NK65 GFP [84] parasites were thawed and passaged once through C57BL/6 mice before being used to infect experimental animals. Animals were infected via intravenous injection of 1x10^4^ parasitised red blood cells (pRBCs). Peripheral parasite burdens of infected mice were followed from day 3 post infection (p.i.) by microscopic examination of giemsa stained thin blood smears and weight loss was monitored. The development of ECM was assessed using a well-established clinical scale [41]: 1 = no signs; 2 = ruffled fur and/or abnormal posture; 3 = lethargy; 4 = reduced responsiveness to stimulation and/or ataxia and/or respiratory distress/hyperventilation; 5 = prostration and/or paralysis and/or convulsions[41]. Stages 4–5 were classified as ECM. *P*.*berghei* ANKA infected mice were euthanised (exposure to a rising concentration of CO_2_) when they reached stage 5 (typically day 7 p.i.) and *P*.*berghei* NK65 mice were culled at the equivalent time point.

### Tissue processing

After termination, the hepatic portal vein was severed and mice were transcardially perfused with 10mls of 0.1M ice cold phosphate buffered saline (PBS) followed by 10mls of ice cold 4% paraformaldehyde (PFA). Brains were dissected out and post-fixed in PFA/20% sucrose for 16-24h at 4°C. Brains were subsequently cryoprotected in PBS/20% sucrose for 48h, snap-frozen in powdered dry ice and stored at -80°C.

Brains were serially sectioned at a thickness of 30μm on a freezing sledge microtome (Bright Instruments, Cambridge, UK). Series of coronal sections encompassing 10 spatially-defined anatomical regions of the brain **(S1)** were stored in cryoprotectant solution (30% ethylene glycol, 20% glycerol in PBS) in the individual wells of a 24 well tissue culture plate (Corning, NY, US) at -20°C until use.

### Immunofluorescence

Brain sections were stained with direct and indirect immunofluorescent technique for the following: i) anti-GFP (1:400 rabbit anti-GFP Alexa Fluor 488 conjugated clone A-21311 Life Technologies) for detection of GFP-tagged parasites; ii) CD31 (1:200 rat monoclonal antibody [mAb] clone MEC 13.3 BD Pharmingen) for visualisation of vasculature; iii) CD3 (1:100 rat mAb clone CD3-12 AbD Serotec) for assessment of cerebral T lymphocyte accumulation; iv) *Lycopersicon esculentum* (tomato) lectin (1:100 [reconstituted 1mg/ml in PBS] biotin conjugated clone L0651 Sigma-Aldrich) for visualisation of vasculature and activated cerebral macrophage populations; v) cleaved caspase 3 (1:200 rabbit mAb clone Asp175 5A1E Cell Signalling) for assessment of cellular apoptosis; vi) NeuN (1:200 mouse mAb biotin conjugated clone A60 Chemicon/Merck Millipore) for demonstration of neuronal architecture; and vii) β-Amyloid Precursor Protein (β-APP 1:200 rabbit polyclonal antibody [pAb] clone CT695 Zymed/Thermo Fisher Scientific) for detection of axonal injury.

The nature of the immunofluorescent staining protocol varied depending on the antibody/epitope pairing. Some stains (i and ii) were performed utilising free-floating protocols. The remaining stains were performed after floating sections were washed in PBS and mounted in distilled water onto Superfrost Plus slides (VWR), before drying vertically overnight at 37°C. All sections were re-hydrated in several changes of PBS before being subjected to heat-mediated antigen retrieval in either Sodium Citrate pH9 buffer (i and ii), Sodium Citrate pH6 buffer (iv, v, vi and vii) or Tris EDTA pH9 buffer (iii, iv and v) pre-heated, respectively, to 80°C, 95°C and 99°C in a water-bath. Sections were subsequently heated for 30 minutes and then allowed to cool at room temperature for 20 minutes. With respect to β-APP (vii), slides were additionally treated with 90% formic acid in distilled water for 15 minutes. All sections were then washed in several changes of wash buffer (0.1M Tris-HCL pH7.5, 0.15M NaCl, 0.05% Tween in distilled water) before being blocked for 1.5 hours at room temperature in block buffer (0.1M Tris-HCL pH7.5, 0.15M NaCl, 1% Bovine Serum Albumin [BSA] 0.3% Triton X in distilled water). Block was removed and sections were incubated with primary antibody diluted appropriately in block buffer; either at room temperature for 3 hours (iv and v) or 12–20 hours at 4°C (i, ii, iii, iv, v, vi and vii). Sections were rinsed several times in wash buffer, and, for fluorescent detection, sections were incubated for 1.5 hours at room temperature in excess quantities of secondary antibodies (goat-anti rat 546, goat anti-rat 647, goat anti-rabbit 546, streptavidin 546, and streptavidin 647 Life Technologies/Thermo Fisher Scientific) diluted in block buffer. An intermediate incubation utilising a biotinylated goat anti-rabbit antibody (Vector) was undertaken to amplify signal for adequate fluorescent visualisation of β-APP (vii), whilst a Tyramide Signal Amplification kit (Thermo Fisher Scientific) was utilised as per manufacturer’s instructions in order to enhance detection of CD3 (iii). Sections were finally washed in several changes of wash buffer and counterstained in DAPI (Sigma-Aldrich). Sections were sequentially rinsed in PBS and distilled water, dried overnight in the dark at room temperature and then coverslipped in ProLong Diamond anti-fade Mountant (Life Technologies).

Antiserum to *Pb* ANKA and *Pb* NK65 infected erythrocytes was prepared as previously described [85]. In brief, mice underwent three rounds of infection and drug cure before whole serum was extracted and IgG purified on Protein-G (HiTrap). Mounted brain sections were blocked with rat serum prior to incubation with anti-*Pb* ANKA or *Pb* NK65 IgG for 1 hr at room temperature. Following incubation with anti-PbA IgG, slides were visualised using FITC rat anti-mouse antibody (clone 11-4011-85: E-Bioscience).

### Immunohistochemistry

To assess BBB permeability, brain sections were stained via indirect immunoperoxidase technique for endogenous Immunoglobulin G (IgG 1:500 Horse pAb biotin conjugated clone BA-2000 Vector). Free-floating sections were washed in PBS and mounted on to Superfrost Plus slides (VWR) in distilled water, then allowed to dry vertically overnight at 37°C. Sections were rehydrated in several changes of PBS and subjected to heat-mediated antigen retrieval in preheated Sodium Citrate pH6 buffer at 95°C for 30 minutes, and then allowed to cool at room temperature for 20 minutes. Slides were rinsed twice in PBS and then endogenous peroxidase activity was blocked by incubation in 3% H^2^O^2^ in distilled water at room temperature for 30 minutes. Sections were washed in several changes of PBS and then incubated for 3 hours at room temperature with primary antibody appropriately diluted in PBS, 0.3% Triton X and 0.1% BSA. Slides were washed thoroughly in PBS and 0.1% tween and then incubated for 1.5 hours at room temperature in ABC solution, as per manufacturer’s instructions (Vector). Colour was developed via a 5 minute incubation in diaminobenzidine tetrahydrochloride (DAB, Merck Millipore). Sections were counterstained with haematoxylin (Vector), dehydrated through alcohol, cleared in two changes of xylene and coverslipped using DPX mounting agent (Sigma-Aldrich).

### Histology

Brain sections were stained via haematoxylin and eosin (H&E) to assess the degree of haemorrhage, parasite sequestration, oedema and white matter disruption. In brief: free-floating sections were washed in PBS and mounted on Superfrost Plus slides (VWR) in distilled water, then allowed to dry vertically overnight at 37°C. Sections were stained using a Thermo Shandon Linstain GLX (Rankin Biomed, US) automated staining machine. Slides were coverslipped using DPX mounting agent (Sigma-Aldrich).

### Brain smears

Animals were terminated via exposure to a rising concentration of CO_2_. The hepatic portal vein was severed and mice transcardially perfused with 10mls PBS. Brains were removed and anatomically comparable regions of cortical and cerebellar grey matter, measuring no greater than 1mm in diameter, were excised. Smears were generated between two microscope slides as previously described [43]. For H&E staining, slides were immediately wet-fixed in acetic alcohol at 4°C, and staining performed as previously described for cytological specimens [86]. For Quik-Diff staining, slides were air-dried before subsequent fixation and staining, performed as per manufactures instructions (Baxter).

### Whole brain homogenate

Animals were terminated via exposure to a rising concentration of CO_2_. The hepatic portal vein was severed and mice transcardially perfused with 10mls PBS. Brains were removed and a single-cell suspension was generated by homogenising tissue through a 70μm cell strainer (BD Falcon) in 5ml of ice-cold PBS. 10μl of cells were pipetted onto a microscope slide and cover-slipped.

### Microscopy

Images were collected on a Zeiss Axioskop upright microscope or Olympus BX51 upright microscope using a 20x objective and captured using a Coolsnap ES camera (photometrics) through MetaVue software (Molecular Devices). Images were then analysed and processed utilising either ImageJ or Image-Pro Premier software (Media Cybernetics).

### Transmission electron microscopy

Under isoflurane anaesthesia, mice were sequentially perfused intracardially with PBS and fixative (2% PFA and 2.5% glutaraldehyde) at 10ml/minute for 7 minutes. Brains were removed and post-fixed for a further 4 hours before anatomically comparable regions of cortical and cerebellar grey matter, measuring approximately 1mm in width, were excised and post-fixed for a further 20 hours. Tissue was then additionally fixed on ice for 1 hour with 1.5% potassium ferrocyanide and 2% osmium tetroxide (weight/vol) in 0.1M cacodylate buffer. This was followed by incubations with 1% thiocarbohydrazide for 20 minutes at room temperature, 2% osmium tetroxide for 30 minutes at room temperature and 1% uranyl acetate at 4°C overnight. The next day, samples were stained with freshly prepared Walton’s lead aspartate (0.02M in lead nitrate and 0.03M in aspartic acid, pH 5.5) for 30 minutes and embedded in Epon 812 (hard forumalation) epoxy resin (Electron Microscopy Science, UK). Resin-embedded samples were subsequently cut at a thickness of 70nm using an ultramicrotome (Leica). Sections were mounted on formvar-coated grids and viewed on an FEI Tecnai 12 Biotwin Transmission Electron Microscope. To assess endothelial cell morphology in each specimen, images of the first 15 capillaries identified were collected digitally using a Gatan Orius SC1000 camera. To examine the interaction between the endothelium and sequestered pRBCs, entire grids were examined at low power to identify regions of interest and then imaged digitally at a high power (Gatan Orius SC1000 camera). Images were analysed and processed utilising ImageJ.

### Data analysis

Ten spatially-defined anatomical regions of the brain, determined by the Allen reference atlas for the C57BL/6 brain [87], were selected for examination. For quantitative purposes 10 random fields of view per region were captured. Distribution, number and/or area of GFP+ parasites, CD3+ T-cells, haemorrhages, extravascular IgG+ permeable vessels and CD31+ vessels were counted manually in a blinded fashion or via ImagePro Premier’s smart segmentation technology in a semi-automated fashion as previously described [26]. Data are expressed as number or area of objects/mm^2^ in a given brain region, or, alternatively, as % of a given distribution within the total number of objects within a given brain region. All statistical analyses were performed using GraphPad PRISM (GraphPad Software). Comparison between two groups was made using unpaired t tests with Welch’s correction. Comparison between multiple groups was made using a one-way ANOVA with Tukey’s test for multiple comparisons. Correlation between different variables within individual brain regions was determined using Spearman-Rank test. Generalised linear models were fitted to the data using the *lm* function of the R statistical language. Linear models were fitted in turn to each measured variable and combinations thereof, within individual brain regions. The quality of different models was compared by computing the R^2^ value.

## Supporting information

S1 Fig Key detailing the ten assessed brain regions. Coronal brain sections stained by H&E (left panel) and with DAPI (right panel), dashed black line delineates each brain region assessed. *Caudoputamen is the rodent equivalent of the Striatum.
(TIF)

S2 Fig Immunofluorescent staining of intracerebral GFP+ parasites in *Pb* ANKA and *Pb* NK65 infected mice and uninfected mice. C57/BL6 mice were infected with 1x10^4^ *Pb* ANKA GFP or *Pb* NK65 GFP pRBCs (n = 6 / group), or left uninfected (n = 5). Mice were culled on d7 p.i. when *Pb* ANKA infected mice exhibited signs of late-stage ECM. Brains were removed from transcardially perfused mice and examined via immunofluorescence for the presence of GFP+ parasite (green) in relation to CD31+ vasculature (red), with nuclei counterstained blue. **(A)** Representative images show the presence of GFP+ parasites (Δ) in identified brain regions of *Pb* ANKA infected mice, and respective absence in *Pb* NK65 infected mice. (Left panel) Magnified view of identified parasite. **(B)** Representative images show absence of GFP (green) in brain regions of uninfected mice. Scale bar: 25μm.
(TIF)

S3 Fig Visualisation of life-cycle stages of intracerebral GFP+ parasites *Pb* ANKA infected mice. C57/BL6 mice were infected with 1x10^4^ *Pb* ANKA GFP pRBCs (n = 3), and culled on d7 p.i. when infected mice exhibited signs of late-stage ECM. Brains were removed from transcardially perfused mice and single-cell suspensions generated for microscopic examination. GFP fluorescence observed in different life cycle stages of the parasite seen in the brains of *Pb* ANKA infected mice.
(TIF)

S4 Fig Immunofluorescent staining of *Pb* anti-sera+ parasite material in the brains of *Pb* ANKA and *Pb* NK65 infected mice. C57/BL6 mice were infected with 1x10^4^ *Pb* ANKA GFP or *Pb* NK65 GFP pRBCs (n = 5 / group), and culled on d7 p.i. when *Pb* ANKA GFP infected mice exhibited signs of late-stage ECM. Brains were removed from transcardially perfused mice and examined via immunofluorescence for the presence of *Pb* anti-sera+ parasite material (green). (N = 5 / group).
(TIF)

S5 Fig T-cells isolated from the whole brain of *Pb* ANKA infected mice at d7 p.i. are mainly CD8^+^. C57/BL6 mice were infected with 1x10^4^ *Pb* ANKA GFP (n = 5). Mice were culled on d7 p.i. when *Pb* ANKA infected mice exhibited signs of late-stage ECM. Whole brains were removed from transcardially perfused mice and processed for flow cytometry. Representative flow plots showing the frequency of CD4^+^ and CD8^+^ cells after gating on CD45^high^ CD11b^dim^ (lymphocytes).
(TIF)

S6 Fig Immunofluorescent staining of intracerebral CD3+ T-cells in *Pb* ANKA and *Pb* NK65 infected mice and uninfected mice. C57/BL6 mice were infected with 1x10^4^ *Pb* ANKA GFP or *Pb* NK65 GFP pRBCs (n = 5 / group), or left uninfected (n = 4). Mice were culled on d7 p.i. when *Pb* ANKA infected mice exhibited signs of late-stage ECM. Brains were removed from transcardially perfused mice and examined via immunofluorescence for the presence of CD3+ T-cells (green) in relation to lectin+ macrophages and vasculature (red), with nuclei counterstained blue. **(A)** Representative images show the presence of CD3+ T-cells in the specified brain regions of *Pb* ANKA and *Pb* NK65 infected mice. **(B)** Representative images show absence of CD3+ T-cells (green) in the specified brain regions of uninfected mice. Scale bar: 25μm.
(TIF)

S7 Fig Immunofluorescent staining detailing leukocyte composition of cerebral packed vessels in *Pb* ANKA infected mice, and histological staining and associated quantification of lymphocyte and pRBC co-localisation in *Pb* ANKA infected mice. C57/BL6 mice were infected with 1x10^4^ *Pb* ANKA GFP pRBCs (n = 5 / group). Mice were culled on d7 p.i. when they exhibited signs of late-stage ECM. Brains were removed following transcardial perfusion and examined via immunofluorescence for the presence of CD3+ T-cells (green) in relation to lectin+ macrophages and vasculature (red), with nuclei counterstained blue. Representative images show: **(A)** larger calibre vessel packed with leukocytes, (Δ) lectin+ macrophages can be seen in the bend of the vessel, with remaining leukocytes (unlabelled) likely monocytes; and **(B)** Lectin+ macrophages and CD3+ T-cells observed in the same distended vessel. Scale bar: 25μm. C57/BL6 mice were infected with 1x10^4^ *Pb* ANKA GFP pRBCs (n = 5 / group). Mice were culled on d7 p.i. when they exhibited signs of late-stage ECM. Brains were removed following transcardial perfusion, smeared for cytological examination and stained by H&E. All pRBCs and lymphocytes (morphologically defined) from 120 total vessels were assessed. Lymphocytes and pRBCs were defined as co-localised if they were associated with the same vessel and within 10μm of one another. If they did not fulfil these criteria they were classed as independent. Representative images showing: **(A)** a pRBC (▲) independent of lymphocytes; **(B)** independent lymphocyte, **(C)** pRBC and T-cell co-localised. **(D)** Quantitation showing relative frequency of independent or co-localised lymphocytes and pRBCs.
(TIF)

S8 Fig H&E staining of haemorrhage in *Pb* ANKA and *Pb* NK65 infected mice and uninfected mice. C57/BL6 mice were infected with 1x10^4^ *Pb* ANKA GFP (n = 8) or *Pb* NK65 GFP pRBCs (n = 5), or left uninfected (n = 5). Mice were culled on d7 p.i. when *Pb* ANKA infected mice exhibited signs of late-stage ECM. Brains were removed from transcardially perfused mice and examined histologically via H&E for haemorrhage. **(A)** Representative images show the presence of haemorrhage in the brain regions of *Pb* ANKA infected mice, and respective absence in *Pb* NK65 infected mice. **(B)** Representative images show the absence of haemorrhage in the brain regions of uninfected mice. Scale bar: 75μm.
(TIF)

S9 Fig Immunohistochemical staining of intracerebral IgG in *Pb* ANKA and *Pb* NK65 infected mice. C57/BL6 mice were infected with 1x10^4^ *Pb* ANKA GFP or *Pb* NK65 GFP pRBCs (n = 5 / group), or left uninfected (n = 5). Mice were culled on d7 p.i. when *Pb* ANKA infected mice exhibited signs of late-stage ECM. Brains were removed from transcardially perfused mice and examined via immunohistochemistry for presence of IgG. Representative images show the higher prevalence of endogenous IgG in the specified brain regions in *Pb* ANKA compared with *Pb* NK65 infected mice. Scale bar: 75μm.
(TIF)

S10 Fig Immunohistochemical staining of intracerebral IgG in uninfected mice. Transcardially perfused brains were removed from uninfected mice and examined via immunohistochemistry for the presence of IgG. Representative images show the absence of endogenous IgG in the specified brain regions of uninfected mice. Scale bar: 75μm.
(TIF)

S11 Fig Immunofluorescent staining of intracerebral cleaved caspase 3+ apoptotic cells in *Pb* ANKA and *Pb* NK65 infected mice and uninfected mice. C57/BL6 mice were infected with 1x10^4^ *Pb* ANKA GFP or *Pb* NK65 GFP pRBCs (n = 5 / group), or left uninfected (n = 5). Mice were culled on d7 p.i. when *Pb* ANKA infected mice exhibited signs of late-stage ECM. Brains were removed from transcardially perfused mice and examined via immunofluorescence for the presence of CC3+ cells (green) in relation to lectin+ macrophages and vasculature (red), with nuclei counterstained blue. **(A)** Representative images show the presence of CC3+ cells (Δ) in the specified brain regions of *Pb* ANKA infected mice, and their respective absence in *Pb* NK65 infected mice. (Left panel) Magnified view of CC3+ cells. **(B)** Representative images show absence of GFP (green) in specified brain regions in uninfected mice. **(C)** Rare CC3+ cells in the brains of *Pb* NK65 infected mice. Scale bar: A = 75μm; B & C = 25μm.
(TIF)

S12 Fig Immunofluorescent staining and quantitation of CD31+ vasculature in the brains of *Pb* ANKA and *Pb* NK65 infected mice and uninfected mice. C57/BL6 mice were infected with 1x10^4^ *Pb* ANKA GFP or *Pb* NK65 GFP pRBCs (n = 4 / group), or left uninfected (n = 4). Mice were culled on d7 p.i. when *Pb* ANKA infected mice exhibited signs of late-stage ECM. Brains were removed from transcardially perfused mice and examined via immunofluorescence for the presence of CD31+ vasculature (red), with nuclei counterstained blue. **(A)** Representative images show CD31+ vasculature (red) in specified brain regions in *Pb* ANKA and *Pb* NK65 infected mice and uninfected mice. **(B)** Quantification of CD31+ vessel number and area, and degree of vascularity (number*area) within all assessed brain regions in *Pb* ANKA and *Pb* NK65 infected mice and uninfected mice. Bars represent mean for all brains within a group, lines show SD within a group. Tukey’s test for multiple comparisons showed no significant difference between any groups, within any of the assessed brain regions.
(TIF)

S13 Fig Representative transmission electron micrographs of capillaries and small venules in the brains of *Pb* ANKA and *Pb* NK65 infected mice and uninfected mice. C57/BL6 mice were infected with 1x10^4^ *Pb* ANKA GFP or *Pb* NK65 GFP pRBCs (n = 3 / group), or left uninfected (n = 3). Mice were culled on d7 p.i. when *Pb* ANKA infected mice exhibited signs of late-stage ECM. Brains were removed from transcardially perfused mice and examined via TEM. Panels from left to right show representative electron micrographs of small venules and capillaries from the brains of *Pb* ANKA and *Pb* NK65 infected mice, and uninfected mice.
(TIF)

S14 Fig Immunofluorescent co-staining of NeuN+ neurons and CC3+ apoptotic cells in the brains of *Pb* ANKA and *Pb* NK65 infected mice. C57/BL6 mice were infected with 1x10^4^ *Pb* ANKA GFP (n = 7) or *Pb* NK65 GFP pRBCs (n = 5). Mice were culled on d7 p.i. when *Pb* ANKA infected mice exhibited signs of late-stage ECM. Brains were removed from transcardially perfused mice and examined via immunofluorescence for the presence of NeuN and CC3. **(A)** Representative images show the neuronal architecture is broadly unaltered in the brains of mice infected with *Pb* ANKA or *Pb* NK65. **(B)** Rare CC3+ apoptotic cells seen in the brains of *Pb* ANKA infected mice do not co-localise with NeuN (white dashed line denotes vessel). Scale bar: 25μm.
(TIF)

S15 Fig Correlation between different histopathological parameters in the brains of *Pb* ANKA infected mice. The regional correlation between any two histopathological parameters in the brains of mice infected with *Pb* ANKA and exhibiting symptoms of late-stage ECM (n = 4–8). Dots represent the mean of the group within a region. p: * ≤0.05, ** <0.005 (Spearman’s rank correlation coefficient).
(TIF)

S1 Table Generalised linear model analyses predicting the relative contribution of different histopathological parameters alone, and in combination, to vascular leakage in the brains of *Pb* ANKA infected mice. Table ranks models by R^2^ value. R^2^ value determines how closely observed data aligns with values predicted by generalised linear modelling.
(TIF)
