# Supplementary figures and images for: A quantitative brain map of experimental cerebral malaria pathology
Source: PLoS Pathog. 2017 Mar 8;13(3):e1006267. doi: 10.1371/journal.ppat.1006267 (PMC5358898; doi:10.1371/journal.ppat.1006267)

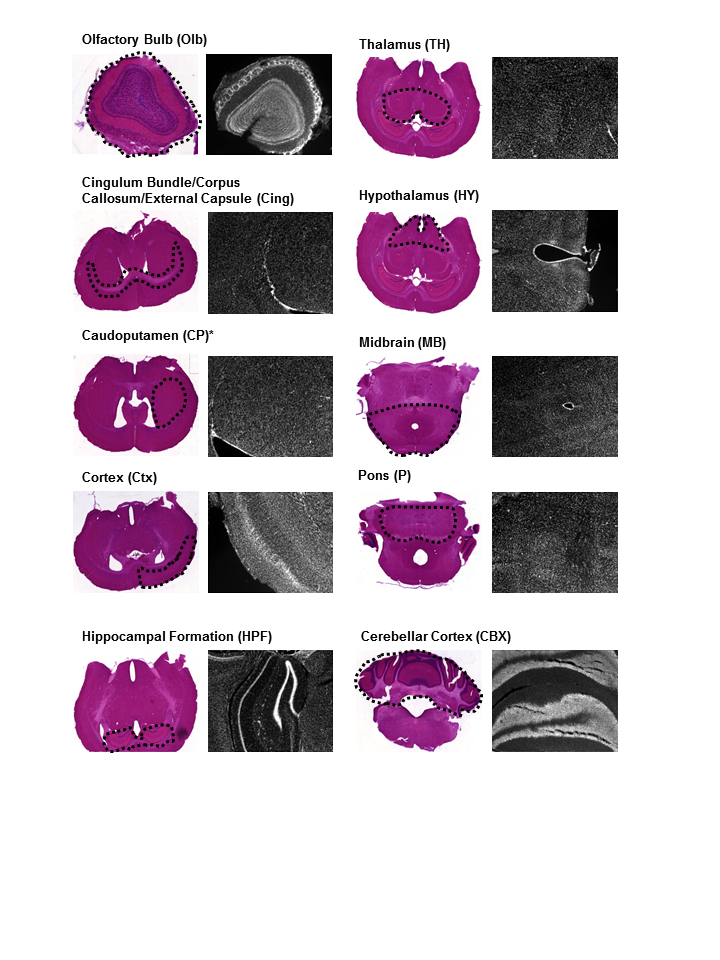

Supplement: S1 Fig — Coronal brain sections stained by H&E (left panel) and with DAPI (right panel), dashed black line delineates each brain region assessed. *Caudoputamen is the rodent equivalent of the Striatum. (TIF) [file ppat.1006267.s001.tif]

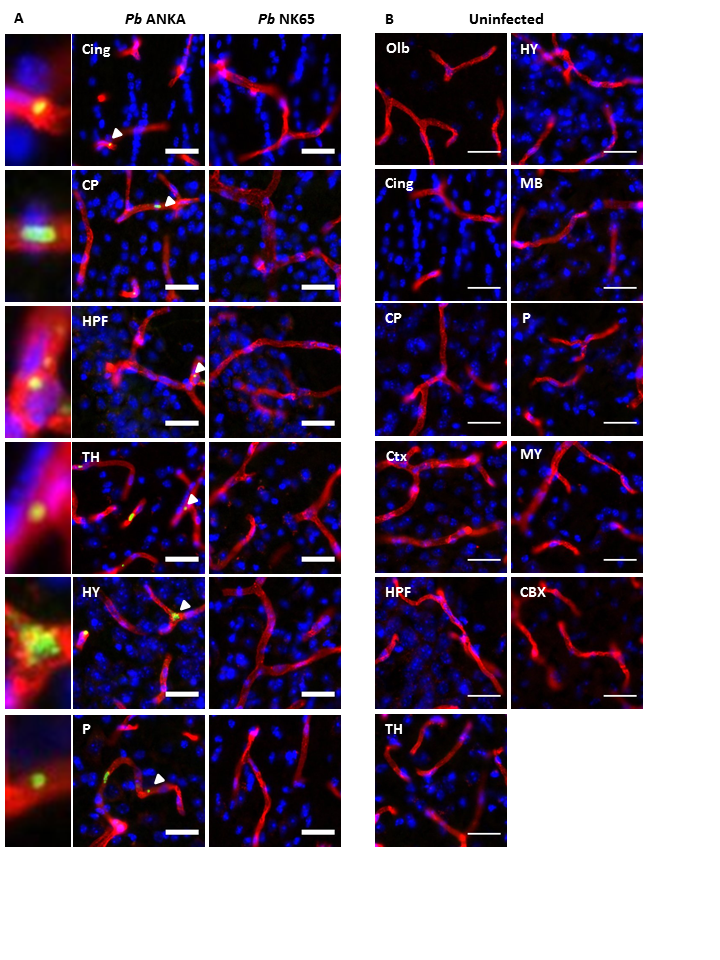

Supplement: S2 Fig — C57/BL6 mice were infected with 1x10^4^ Pb ANKA GFP or Pb NK65 GFP pRBCs (n = 6 / group), or left uninfected (n = 5). Mice were culled on d7 p.i. when Pb ANKA infected mice exhibited signs of late-stage ECM. Brains were removed from transcardially perfused mice and examined via immunofluorescence for the presence of GFP+ parasite (green) in relation to CD31+ vasculature (red), with nuclei counterstained blue. (A) Representative images show the presence of GFP+ parasites (Δ) in identified brain regions of Pb ANKA infected mice, and respective absence in Pb NK65 infected mice. (Left panel) Magnified view of identified parasite. (B) Representative images show absence of GFP (green) in brain regions of uninfected mice. Scale bar: 25μm. (TIF) [file ppat.1006267.s002.tif]

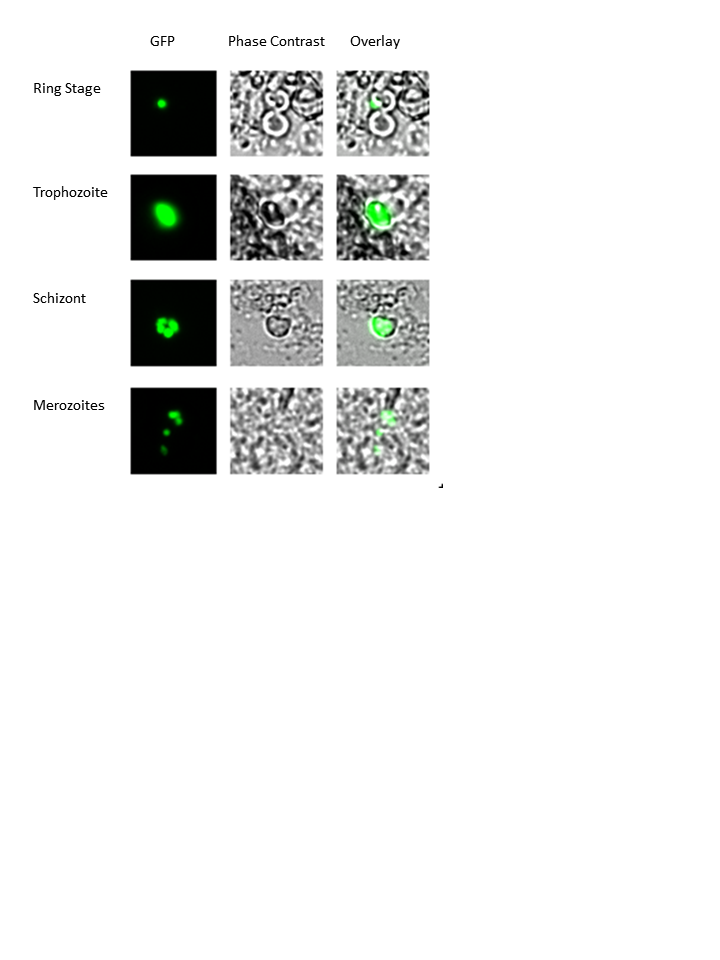

Supplement: S3 Fig — C57/BL6 mice were infected with 1x10^4^ Pb ANKA GFP pRBCs (n = 3), and culled on d7 p.i. when infected mice exhibited signs of late-stage ECM. Brains were removed from transcardially perfused mice and single-cell suspensions generated for microscopic examination. GFP fluorescence observed in different life cycle stages of the parasite seen in the brains of Pb ANKA infected mice. (TIF) [file ppat.1006267.s003.tif]

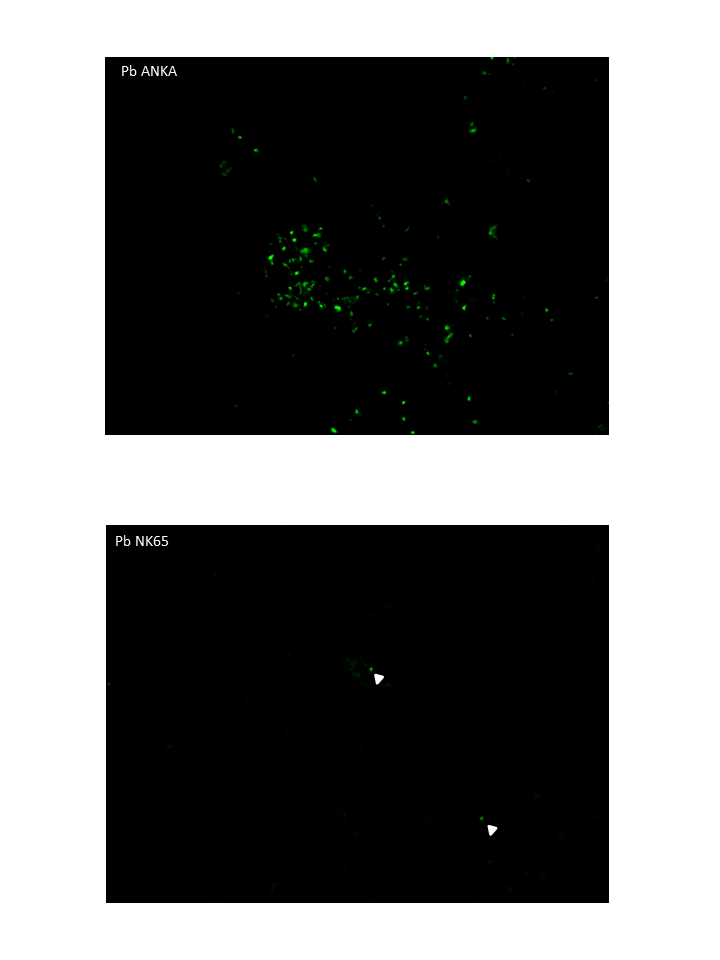

Supplement: S4 Fig — C57/BL6 mice were infected with 1x10^4^ Pb ANKA GFP or Pb NK65 GFP pRBCs (n = 5 / group), and culled on d7 p.i. when Pb ANKA GFP infected mice exhibited signs of late-stage ECM. Brains were removed from transcardially perfused mice and examined via immunofluorescence for the presence of Pb anti-sera+ parasite material (green). (N = 5 / group). (TIF) [file ppat.1006267.s004.tif]

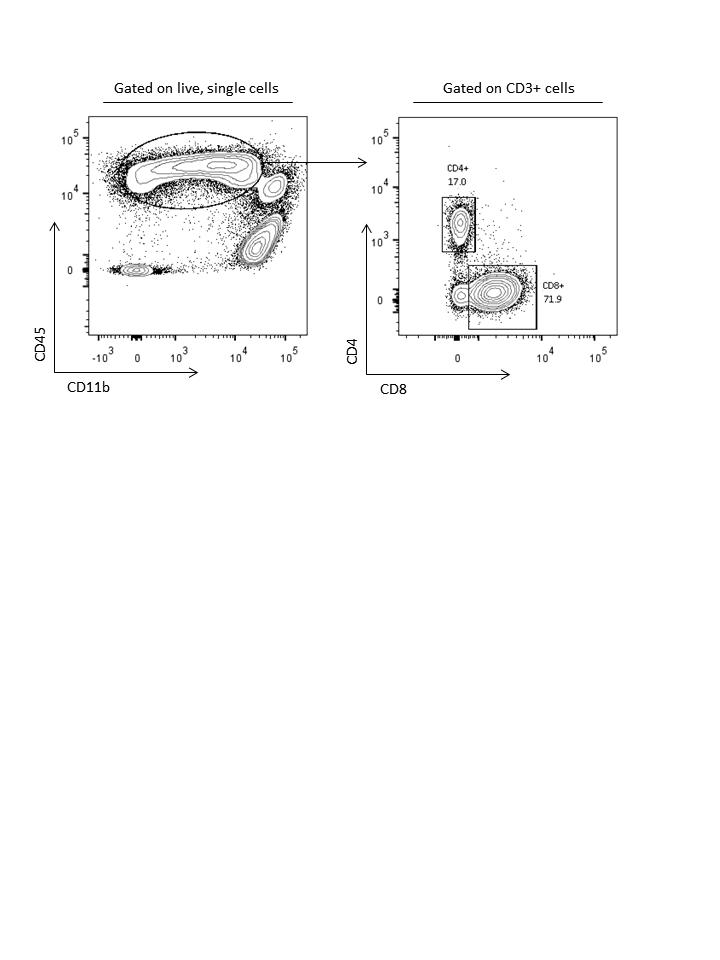

Supplement: S5 Fig — C57/BL6 mice were infected with 1x10^4^ Pb ANKA GFP (n = 5). Mice were culled on d7 p.i. when Pb ANKA infected mice exhibited signs of late-stage ECM. Whole brains were removed from transcardially perfused mice and processed for flow cytometry. Representative flow plots showing the frequency of CD4+ and CD8+ cells after gating on CD45high CD11bdim (lymphocytes). (TIF) [file ppat.1006267.s005.tif]

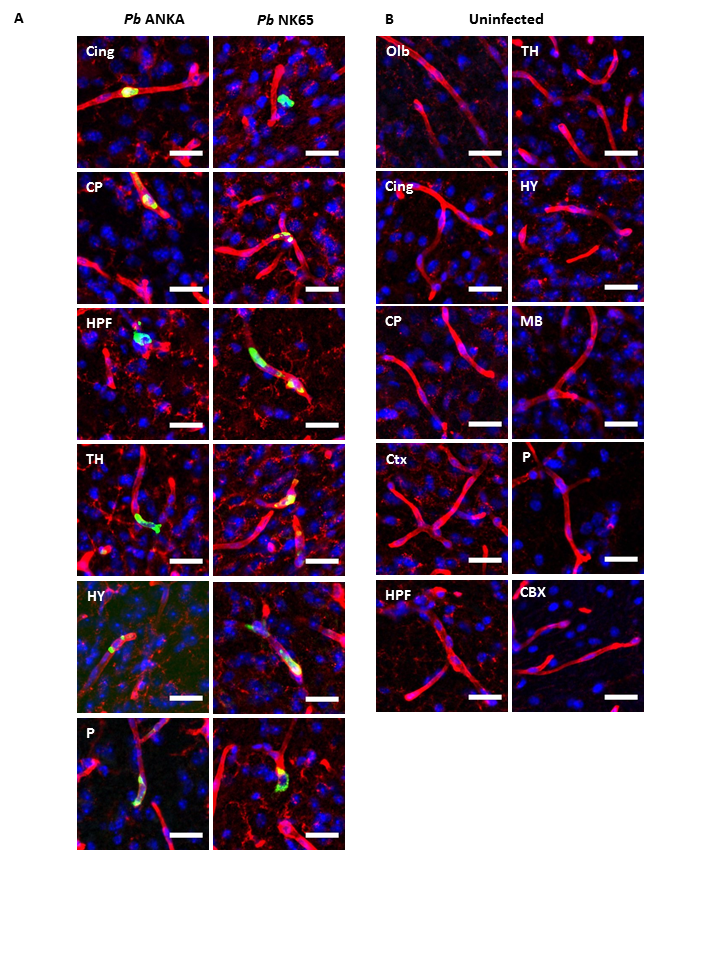

Supplement: S6 Fig — C57/BL6 mice were infected with 1x10^4^ Pb ANKA GFP or Pb NK65 GFP pRBCs (n = 5 / group), or left uninfected (n = 4). Mice were culled on d7 p.i. when Pb ANKA infected mice exhibited signs of late-stage ECM. Brains were removed from transcardially perfused mice and examined via immunofluorescence for the presence of CD3+ T-cells (green) in relation to lectin+ macrophages and vasculature (red), with nuclei counterstained blue. (A) Representative images show the presence of CD3+ T-cells in the specified brain regions of Pb ANKA and Pb NK65 infected mice. (B) Representative images show absence of CD3+ T-cells (green) in the specified brain regions of uninfected mice. Scale bar: 25μm. (TIF) [file ppat.1006267.s006.tif]

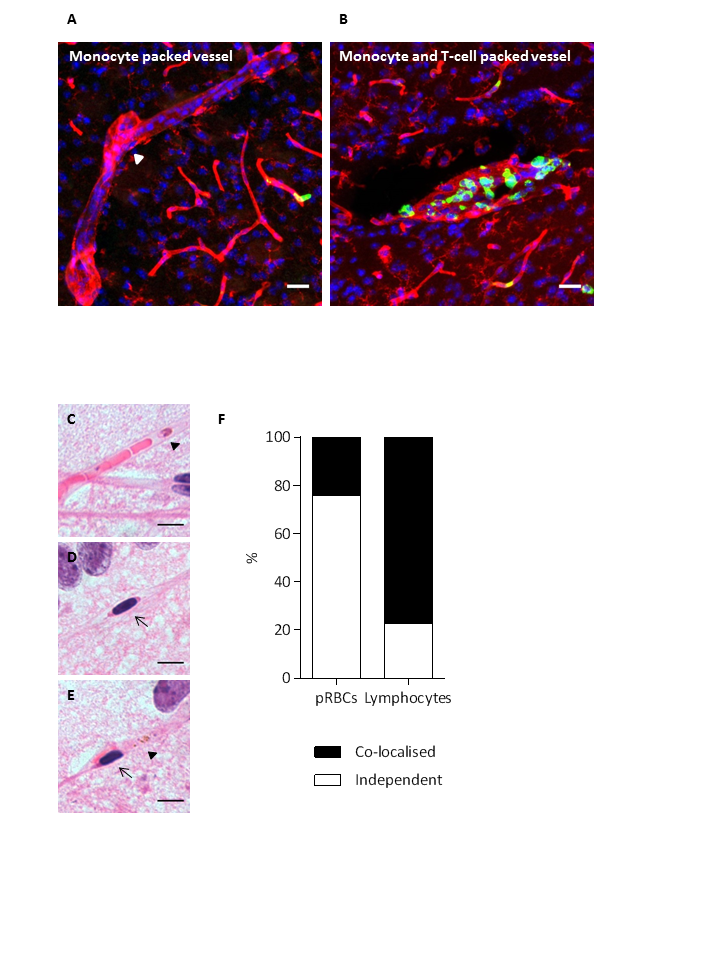

Supplement: S7 Fig — C57/BL6 mice were infected with 1x10^4^ Pb ANKA GFP pRBCs (n = 5 / group). Mice were culled on d7 p.i. when they exhibited signs of late-stage ECM. Brains were removed following transcardial perfusion and examined via immunofluorescence for the presence of CD3+ T-cells (green) in relation to lectin+ macrophages and vasculature (red), with nuclei counterstained blue. Representative images show: (A) larger calibre vessel packed with leukocytes, (Δ) lectin+ macrophages can be seen in the bend of the vessel, with remaining leukocytes (unlabelled) likely monocytes; and (B) Lectin+ macrophages and CD3+ T-cells observed in the same distended vessel. Scale bar: 25μm. C57/BL6 mice were infected with 1x10^4^ Pb ANKA GFP pRBCs (n = 5 / group). Mice were culled on d7 p.i. when they exhibited signs of late-stage ECM. Brains were removed following transcardial perfusion, smeared for cytological examination and stained by H&E. All pRBCs and lymphocytes (morphologically defined) from 120 total vessels were assessed. Lymphocytes and pRBCs were defined as co-localised if they were associated with the same vessel and within 10μm of one another. If they did not fulfil these criteria they were classed as independent. Representative images showing: (A) a pRBC (▲) independent of lymphocytes; (B) independent lymphocyte, (C) pRBC and T-cell co-localised. (D) Quantitation showing relative frequency of independent or co-localised lymphocytes and pRBCs. (TIF) [file ppat.1006267.s007.tif]

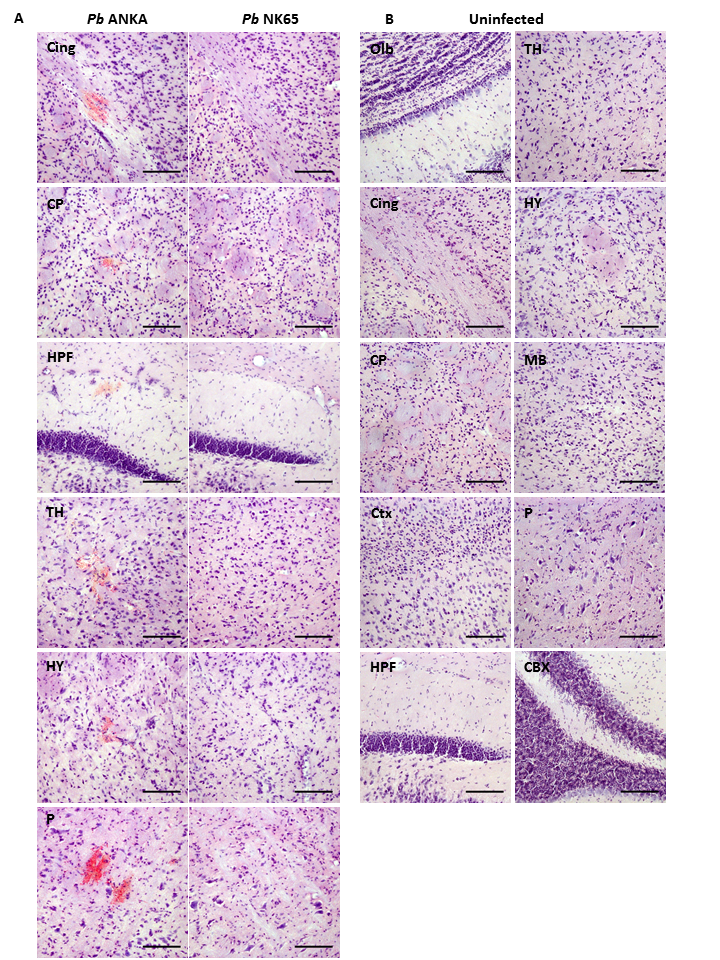

Supplement: S8 Fig — C57/BL6 mice were infected with 1x10^4^ Pb ANKA GFP (n = 8) or Pb NK65 GFP pRBCs (n = 5), or left uninfected (n = 5). Mice were culled on d7 p.i. when Pb ANKA infected mice exhibited signs of late-stage ECM. Brains were removed from transcardially perfused mice and examined histologically via H&E for haemorrhage. (A) Representative images show the presence of haemorrhage in the brain regions of Pb ANKA infected mice, and respective absence in Pb NK65 infected mice. (B) Representative images show the absence of haemorrhage in the brain regions of uninfected mice. Scale bar: 75μm. (TIF) [file ppat.1006267.s008.tif]

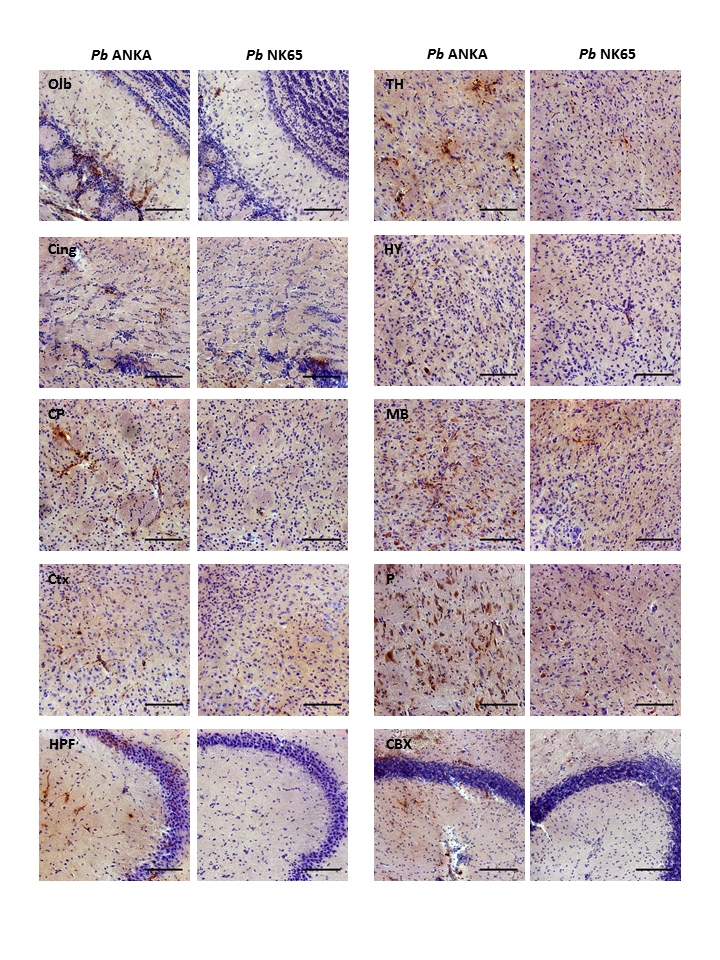

Supplement: S9 Fig — C57/BL6 mice were infected with 1x10^4^ Pb ANKA GFP or Pb NK65 GFP pRBCs (n = 5 / group), or left uninfected (n = 5). Mice were culled on d7 p.i. when Pb ANKA infected mice exhibited signs of late-stage ECM. Brains were removed from transcardially perfused mice and examined via immunohistochemistry for presence of IgG. Representative images show the higher prevalence of endogenous IgG in the specified brain regions in Pb ANKA compared with Pb NK65 infected mice. Scale bar: 75μm. (TIF) [file ppat.1006267.s009.tif]

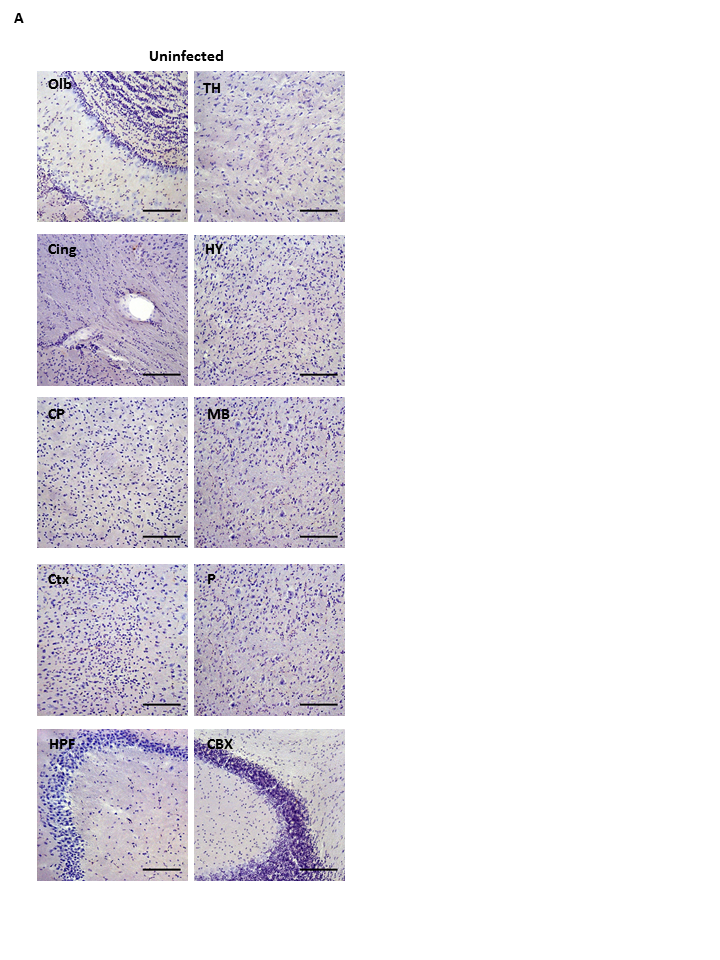

Supplement: S10 Fig — Transcardially perfused brains were removed from uninfected mice and examined via immunohistochemistry for the presence of IgG. Representative images show the absence of endogenous IgG in the specified brain regions of uninfected mice. Scale bar: 75μm. (TIF) [file ppat.1006267.s010.tif]

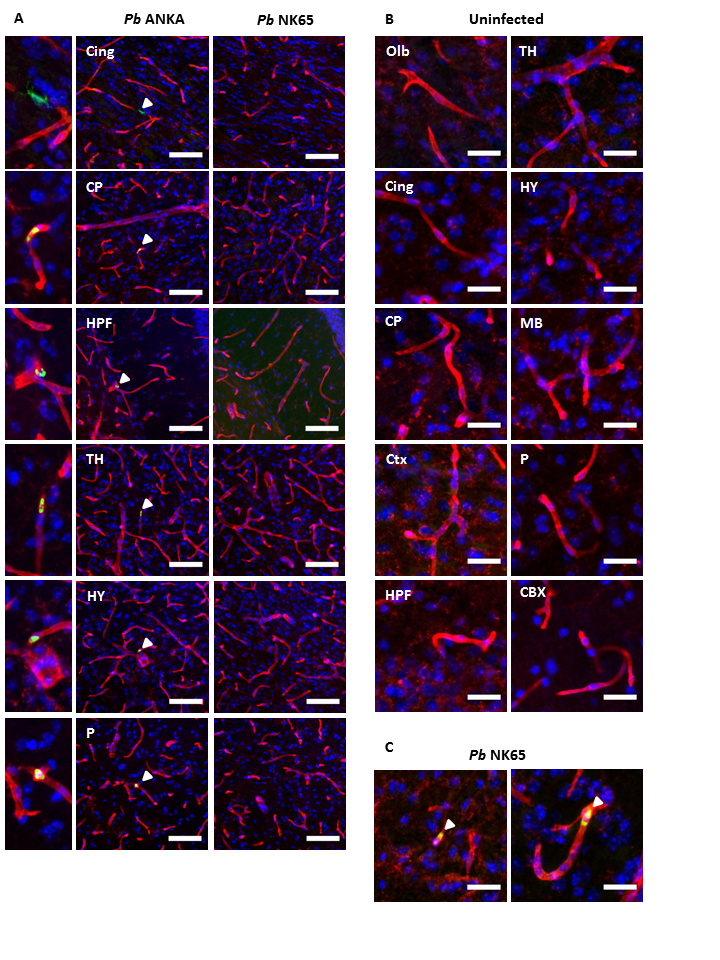

Supplement: S11 Fig — C57/BL6 mice were infected with 1x10^4^ Pb ANKA GFP or Pb NK65 GFP pRBCs (n = 5 / group), or left uninfected (n = 5). Mice were culled on d7 p.i. when Pb ANKA infected mice exhibited signs of late-stage ECM. Brains were removed from transcardially perfused mice and examined via immunofluorescence for the presence of CC3+ cells (green) in relation to lectin+ macrophages and vasculature (red), with nuclei counterstained blue. (A) Representative images show the presence of CC3+ cells (Δ) in the specified brain regions of Pb ANKA infected mice, and their respective absence in Pb NK65 infected mice. (Left panel) Magnified view of CC3+ cells. (B) Representative images show absence of GFP (green) in specified brain regions in uninfected mice. (C) Rare CC3+ cells in the brains of Pb NK65 infected mice. Scale bar: A = 75μm; B & C = 25μm. (TIF) [file ppat.1006267.s011.tif]

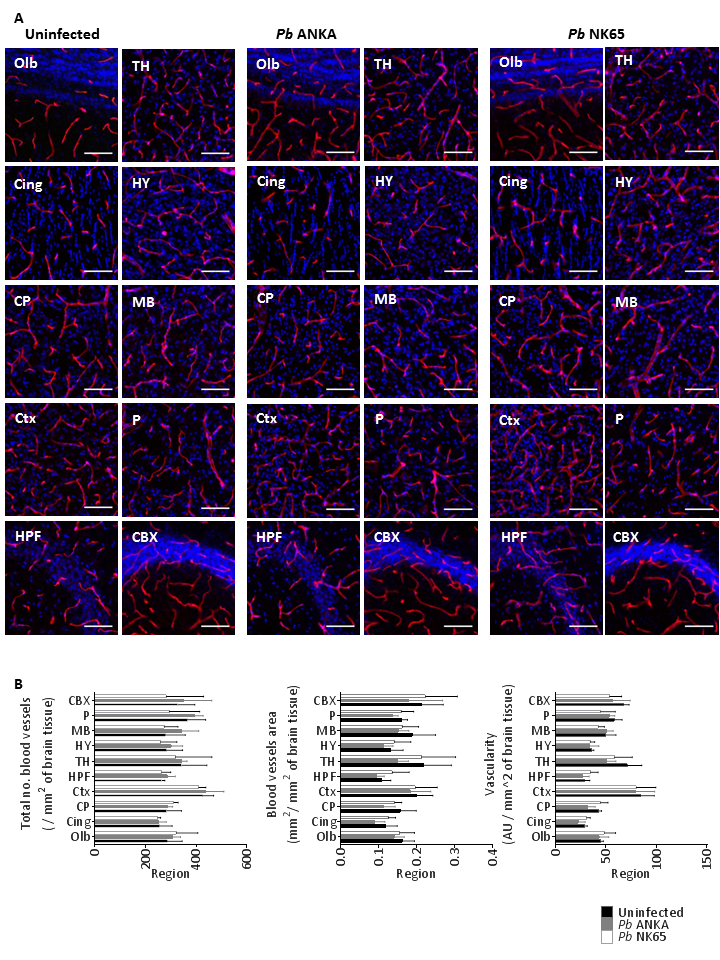

Supplement: S12 Fig — C57/BL6 mice were infected with 1x10^4^ Pb ANKA GFP or Pb NK65 GFP pRBCs (n = 4 / group), or left uninfected (n = 4). Mice were culled on d7 p.i. when Pb ANKA infected mice exhibited signs of late-stage ECM. Brains were removed from transcardially perfused mice and examined via immunofluorescence for the presence of CD31+ vasculature (red), with nuclei counterstained blue. (A) Representative images show CD31+ vasculature (red) in specified brain regions in Pb ANKA and Pb NK65 infected mice and uninfected mice. (B) Quantification of CD31+ vessel number and area, and degree of vascularity (number*area) within all assessed brain regions in Pb ANKA and Pb NK65 infected mice and uninfected mice. Bars represent mean for all brains within a group, lines show SD within a group. Tukey’s test for multiple comparisons showed no significant difference between any groups, within any of the assessed brain regions. (TIF) [file ppat.1006267.s012.tif]

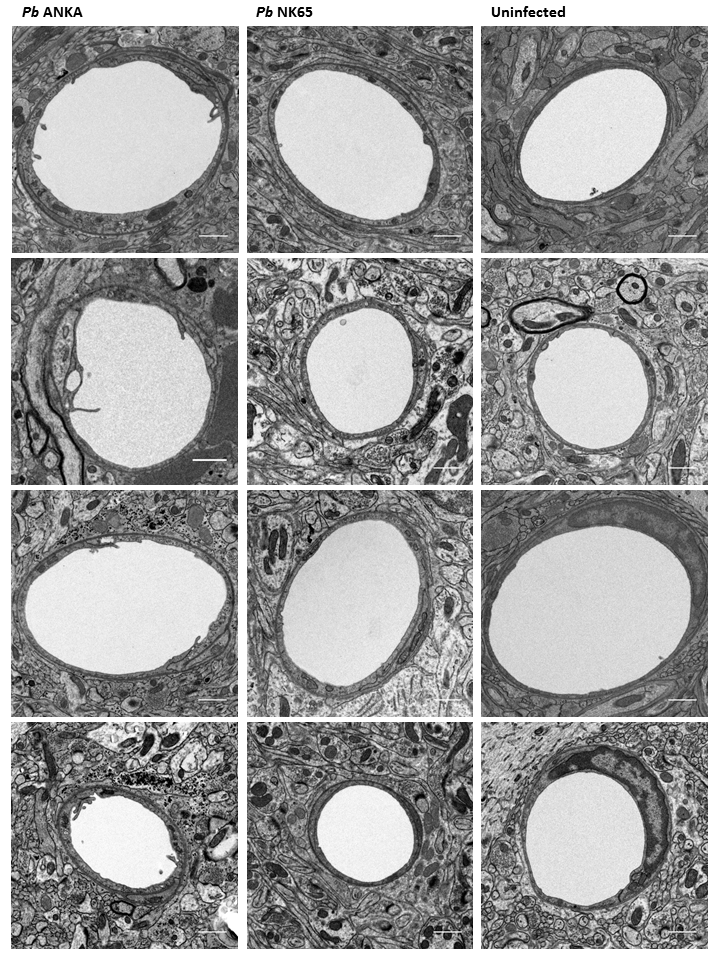

Supplement: S13 Fig — C57/BL6 mice were infected with 1x10^4^ Pb ANKA GFP or Pb NK65 GFP pRBCs (n = 3 / group), or left uninfected (n = 3). Mice were culled on d7 p.i. when Pb ANKA infected mice exhibited signs of late-stage ECM. Brains were removed from transcardially perfused mice and examined via TEM. Panels from left to right show representative electron micrographs of small venules and capillaries from the brains of Pb ANKA and Pb NK65 infected mice, and uninfected mice. (TIF) [file ppat.1006267.s013.tif]

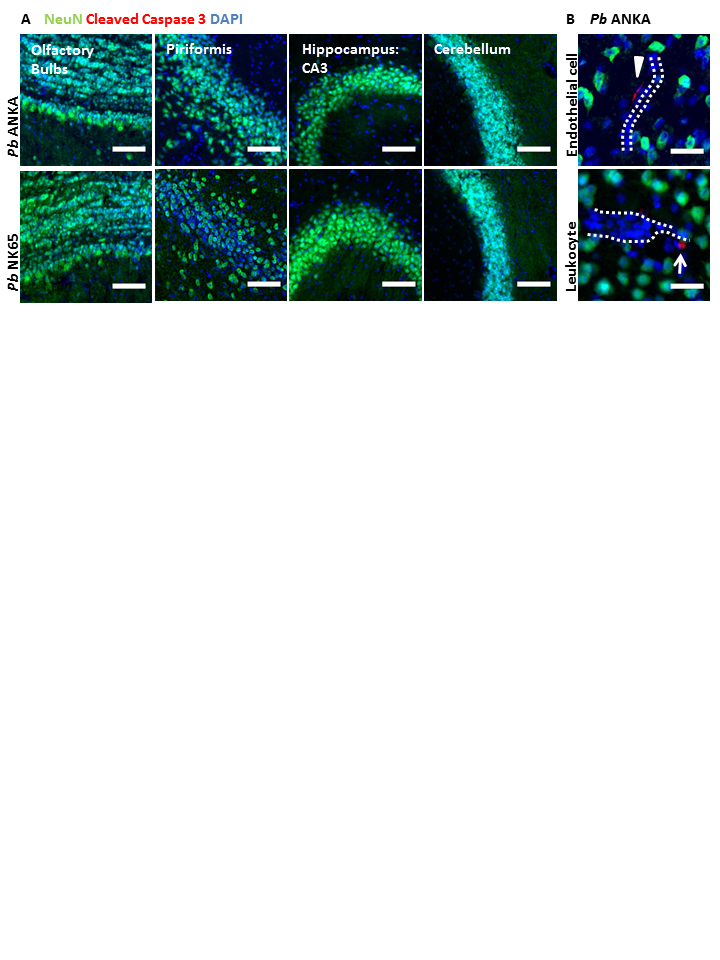

Supplement: S14 Fig — C57/BL6 mice were infected with 1x10^4^ Pb ANKA GFP (n = 7) or Pb NK65 GFP pRBCs (n = 5). Mice were culled on d7 p.i. when Pb ANKA infected mice exhibited signs of late-stage ECM. Brains were removed from transcardially perfused mice and examined via immunofluorescence for the presence of NeuN and CC3. (A) Representative images show the neuronal architecture is broadly unaltered in the brains of mice infected with Pb ANKA or Pb NK65. (B) Rare CC3+ apoptotic cells seen in the brains of Pb ANKA infected mice do not co-localise with NeuN (white dashed line denotes vessel). Scale bar: 25μm. (TIF) [file ppat.1006267.s014.tif]

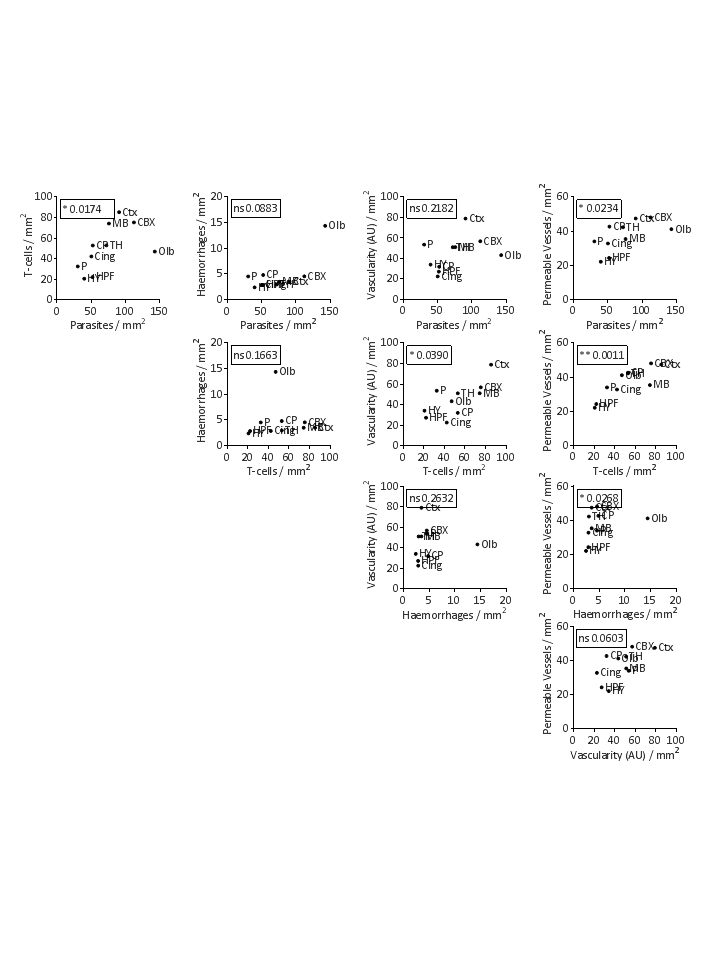

Supplement: S15 Fig — The regional correlation between any two histopathological parameters in the brains of mice infected with Pb ANKA and exhibiting symptoms of late-stage ECM (n = 4–8). Dots represent the mean of the group within a region. p: * ≤0.05, ** <0.005 (Spearman’s rank correlation coefficient). (TIF) [file ppat.1006267.s015.tif]

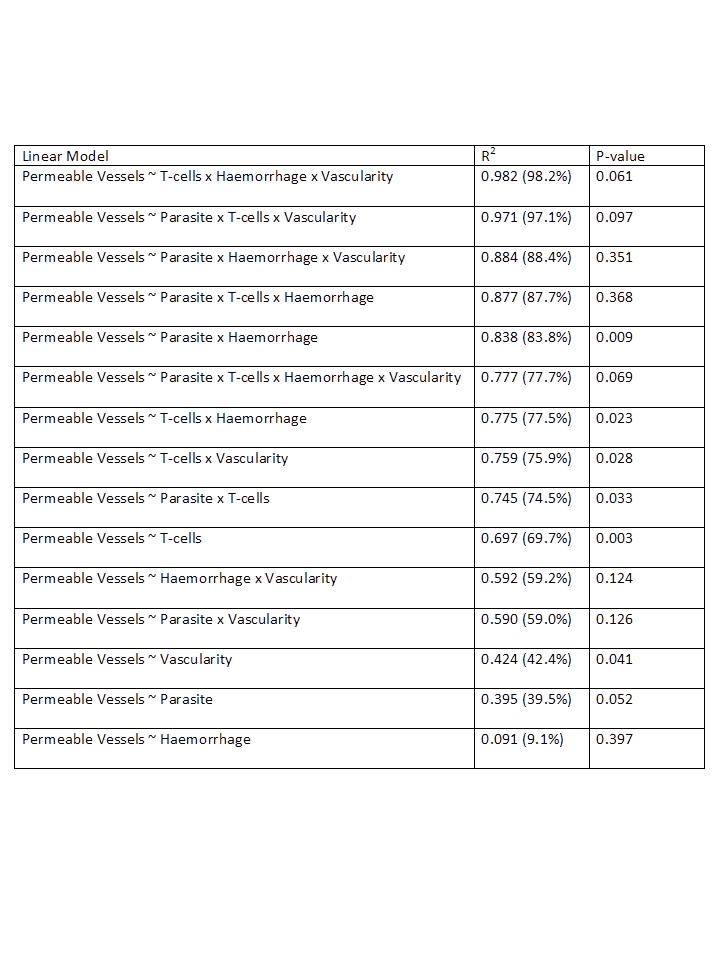

Supplement: S1 Table — Table ranks models by R2 value. R2 value determines how closely observed data aligns with values predicted by generalised linear modelling. (TIF) [file ppat.1006267.s016.tif]
